# DCE-MRI-Derived Parameters in Evaluating Abraxane-Induced Early Vascular Response and the Effectiveness of Its Synergistic Interaction with Cisplatin

**DOI:** 10.1371/journal.pone.0162601

**Published:** 2016-09-15

**Authors:** Xilin Sun, Lili Yang, Xuefeng Yan, Yingying Sun, Dongliang Zhao, Yang Ji, Kai Wang, Xiaoyuan Chen, Baozhong Shen

**Affiliations:** 1 Department of Radiology, the Fourth Hospital of Harbin Medical University, Harbin, Heilongjiang, China; 2 Molecular Imaging Research Center of Harbin Medical University, Harbin, Heilongjiang, China; 3 National Institute of Biomedical Imaging and Bioengineering (NIBIB), National Institutes of Health (NIH), Bethesda, Maryland, United States of America; Kyoto Daigaku, JAPAN

## Abstract

Our previous studies revealed molecular alterations of tumor vessels, varying from immature to mature alterations, resulting from Abraxane, and demonstrated that the integrin-specific PET tracer ^18^F-FPPRGD2 can be used to noninvasively monitor such changes. However, changes in the tumor vasculature at functional levels such as perfusion and permeability are also important for monitoring Abraxane treatment outcomes in patients with cancer. The purpose of this study is to further investigate the vascular response during Abraxane therapy and the effectiveness of its synergistic interaction with cisplatin using Dynamic contrast enhanced-magnetic resonance imaging (DCE-MRI). Thirty MDA-MB-435 tumor mice were randomized into three groups: PBS control (C group), Abraxane only (A group), and sequential treatment with Abraxane followed by cisplatin (A-P group). Tumor volume was monitored based on caliper measurements. A DCE-MRI protocol was performed at baseline and day 3. The K^trans^, K_ep_ and V_e_ were calculated and compared with CD31, α-SMA, and Ki67 histology data. Sequential treatment with Abraxane followed by cisplatin produced a significantly greater inhibition of tumor growth during the three weeks of the observation period. Decreases in K^trans^ and K_ep_ for the A and A-P groups were observed on day 3. Immunohistological staining suggested vascular remodeling during the Abraxane therapy. The changes in K^trans^ and K_ep_ values were correlated with alterations in the permeability of the tumor vasculature induced by the Abraxane treatment. In conclusion, Abraxane-mediated permeability variations in tumor vasculature can be quantitatively visualized by DCE-MRI, making this a useful method for studying the effects of early cancer treatment, especially the early vascular response. Vascular remodeling by Abraxane improves the efficiency of cisplatin delivery and thus results in a favorable treatment outcome.

## Introduction

Abraxane (paclitaxel protein-bound particles for injectable suspension, Abraxis BioScience, Los Angeles, CA, Unite States) is a novel nanoparticle albumin-bound paclitaxel. It has been broadly used to treat advanced (metastatic) breast cancer [1, 2], squamous cell carcinoma of the oropharynx [3], advanced (metastatic) pancreatic cancer, advanced non-small cell lung cancer [4], and other cancers. To further improve the efficacy of weekly Abraxane in advanced (metastatic) cancer patients, combinations of Abraxane with other chemotherapeutic agents or biologics have been tested in several clinical studies [3]. These clinical trials showed that the combination of Abraxane and carboplatin or cisplatin has better short-term efficacy, a rapid therapy response, and manageable toxicity in breast cancer and lung squamous carcinoma [5, 6].

Of particular note is that recent studies investigating the mechanistic aspects of chemotherapy-induced angiogenesis showed that Abraxane can trigger reactionary angiogenesis [7]. This finding provides a strong mechanistic rationale for the combination of Abraxane therapy with carboplatin or cisplatin to enhance the tumor therapy response [7–10]. Because subtle and less predictable effects on tumor hemodynamics may lead to totally different therapeutic results, understanding the full activity of Abraxane in tumor vasculature is of vital importance in developing novel therapeutic strategies and choosing alternative therapies [11].

Our previous studies revealed tumor vessel maturation after Abraxane treatment, and ^18^F-FPPRGD2 (an integrin-specific PET tracer) can noninvasively monitor the integrin α_v_β_3_ level over time [12]. However, the mechanism of Abraxane activity is very complex. The quantification of functional changes in the tumor vasculature, such as perfusion and permeability, is also very important for monitoring the Abraxane treatment outcome in patients with cancer [13]. The functional characteristics of the tumor vasculature are expected to affect the perfusion of contrast agent in the tumor, endothelial permeability and tissue volume fractions [14]. Dynamic contrast enhanced-magnetic resonance imaging (DCE-MRI) has been shown to be sensitive to changes in the physiological characteristics of tumor vasculature by providing quantifiable parameters, such as K^trans^ (the volume transfer coefficient of contrast agent between the extracellular extravascular space (EES) and the blood plasma), K_ep_ (a contrast agent backflux rate constant), Vp (plasma volume fraction), and V_e_ (extravascular extracellular volume fraction). These characteristics potentially enable the use of DCE-MRI to diagnose [15] and manage solid tumors, including therapeutic effect monitoring [16].

To further investigate the effect of Abraxane on angiogenesis and evaluate the therapeutic effects of novel therapeutic strategies, we examined the use of DCE-MRI to assess the vascular response (functional parameters such as permeability and penetration) during Abraxane therapy and the effectiveness of its synergistic interaction with cisplatin.

## Materials and Methods

### Animal and tumor models

Experiments performed in this study were approved by the Harbin Medical University animal ethics committee (Harbin Medical University, Harbin, China) and this study was carried out in strict accordance with the recommendations in the Guide for the Care and Use of Laboratory Animals of the National Institutes of Health.

The MDA-MB-435 cell line was purchased from American Type Culture Collection (ATCC). MDA-MB-435 cells were cultured routinely in Leibovitz’s L-15 medium (Thermo Scientific, Beijing, China) supplemented with 10% Fetal calf serum (FCS, Thermo Scientific) under a 100% air atmosphere at 37°C. All cultures were passaged 2–3 times per week to maintain logarithmic growth. The MDA-MB-435 tumor model was established via the injection of 8×10^6^ cells in the left mammary fat pad of athymic nude mice (female, 6 weeks, SLAC Laboratory Animal Co. LTD, Shanghai, China). Mice were housed in a clean facility with special conditions that include HEPA filtered ventilated cage systems, autoclaved bedding, autoclaved housing, autoclaved, water, irradiated food and special cage changing procedures. Mice were handled under aseptic conditions including the wearing of gloves, gowns and shoe coverings. Tumor diameters (a and b) were measured with clippers three times per week to follow tumor growth. Tumor volume was estimated as (a×b^2^)/2. Animals were monitored during the experimental procedure everyday and were sacrificed when tumors reached 2000 mm^3^. Mice were sacrificed by inhalation of CO2 from a pressurized tank in a mouse chamber. No animals died prior to the experimental endpoint.

### MR imaging of MDA-MB-435 tumor-bearing mice

MR imaging of MDA-MB-435 tumor mice was conducted with a 3.0 T system (Philips, Netherlands) using an animal coil (Philips). During the MRI scans, all animals were anesthetized by inhaled isoflurane (1.5% in O2) at 2.0 LPM. A children’s scalp needle (size: 0.40; KDL Corp., China) was connected to a syringe to deliver gadoteridol (Omniscan^TM^, GE Healthcare, Oslo, Norway). The protocol included anatomical MR imaging as follows: a T2-weighted spin echo sequence (RARE) was performed before contrast agent injection with a field of view = 160 × 116 matrix and 40 × 40 × 26 mm, repetition time (TR)/echo time (TE) = 4,000/100 ms. A slice thickness of 2 mm with a 0.2 mm gap was used to cover the tumor region of interest (ROI). A gradient-echo multiflip-angle T1 map was produced before contrast agent injection with a field of view = 200 × 160 matrix, a 40 × 40 × 26 mm, TR/TE = 651/20 ms, NEX = 3, and flip angles of 10, 20, 30, 40, 50, 60, and 70°. A slice thickness of 2 mm with a 0.2 mm gap was used to cover the tumor ROI. The parameters of DCE-MRI were the same as those above, except the fixed flip angle was 30°. After eight baseline image scans, 0.2 ml/kg of gadoteridol (total injection volume, 0.15 ml) was injected (over a period of 15 s), and then 40 images were acquired.

### Image processing and analysis

**Fig 1:**
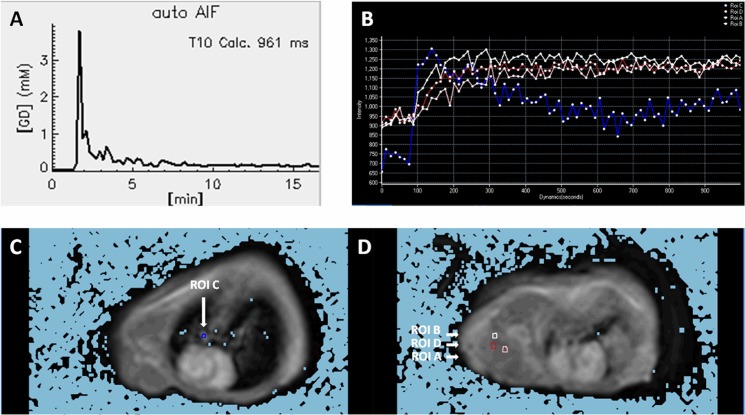
**A representative AIF within the right pulmonary artery** (A) (long white arrow) in (C) using DCE-tool. Four time-intensity curves of the AIF (ROI-C) and tumor ROI (ROI-A, B, D) are shown in (B), using a T1 basic perfusion tool. Their corresponding ROI and size (ROI-A, B, C, D) are given in C and D. A and B demonstrate the consistency of AIF shape fitted with the extended Tolfts model.

DCE-MRI data were analyzed using manufacturer-supplied software (DCE-Tool, Version 5.1, Philips Healthcare, Best, The Netherlands). The main principles were described by Zheng et al. [17]. DCE-MRI data were processed by 3 experienced radiologists. First, a pooled arterial input function (AIF, Fig 1A, Fig 1C), T10 value and Gd concentration were acquired from every mouse in this modeling procedure. Then, the software automatically calculated the resulting output maps, including K^trans^, V_e_, K_ep_. Finally, on the basis of the K^trans^ maps, ROIs covering the primary tumor and large vessels and liquefacient necrotic areas were excluded. Measurements were repeated 3–5 times to reduce the error, and the mean was estimated. The sizes of ROIs and the T10 values were also recorded.

### Experimental design

Thirty mice underwent baseline DCE-MRI (day 0) when the tumors reached a mean volume of 250±60 mm^3^. Then, the tumor-bearing mice were randomly divided into three groups (n = 10/group): C group, a control group receiving physiologic saline on days 0, 2, and 4; A group, intravenous injection of Abraxane at a dose of 25 mg/kg on days 0, 2, and 4; A-P group, pretreatment with Abraxane on days 0, 2, and 4 followed by cisplatin on days 1, 3, and 5. DCE-MRI was repeated on day 3. At each time point, one mouse from each group was sacrificed, and tumors were excised for histopathology.

### Fluorescence staining and histologic analysis

After the mice were euthanized, tumors were harvested and frozen in liquid nitrogen. The tumor was cut into 5 μm thick slices. Samples were stained for mouse anti-human α-SMA (C6198, Sigma), rat anti-mouse CD31 antibody (550389, BD Biosciences), and rabbit anti-mouse Ki76 antibody (180191Z, Invitrogen, Grand Island, NY). All experiments were performed in duplicate and repeated twice. All images were analyzed using Image J software (Imaging Processing and Analysis in Java, NIH image, MD) version 1.48. Image J was used to assess α-SMA fluorescence intensity, vessel number, total DAPI-positive nuclei number, and Ki 67-positive nuclei number. The percentage of positive nuclei within the total number of nuclei was used to define the Ki-67 staining index (SI). For each tumor section, ten random high-power images (200× magnification or 400× magnification) were analyzed.

### Statistical analysis

All quantitative data were expressed as means±SD. Statistical comparisons of sequence-dependent effects were conducted by ANOVA, and P values <0.05 were considered statistically significant.

## Results

### Therapeutic effect of Abraxane and its synergistic interaction with cisplatin on MDA-MB-435 tumors

**Fig 2:**
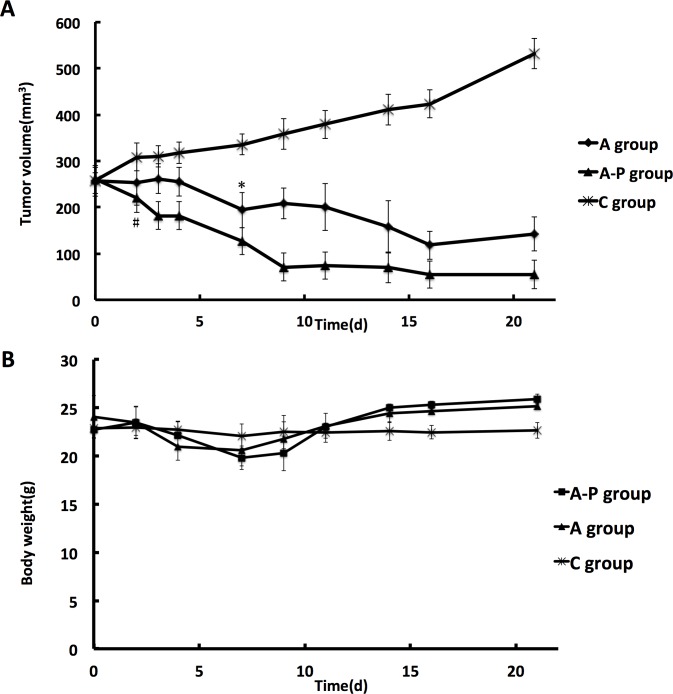
Antitumor effect of Abraxane and combination with Cisplatin in established MDA-MB-435 xenografts. (A) Tumor growth curves under different treatments. *p < 0.05, C group vs. A group. # p < 0.05, C group vs. A-P group. (B) Body weight changes of mice subjected to different treatments.

To assess the function of different therapeutic schedules in inhibiting tumor growth, tumor size and body weight were measured in all groups for 3 weeks. Although all therapeutic schedules resulted in a reduction in tumor volume, the most efficacious against breast cancer was the combined Abraxane and cisplatin therapy. As shown in Fig 2A, for the first four days, Abraxane showed no marked inhibition of tumor growth until day 7. In comparison, the average tumor size of the A-P group became significantly smaller than that of the control group starting from day 2 (P<0.05). Continued tumor growth suppression was observed in the A-P group during the three-week observation period. Mouse body weight was monitored as an indicator of the toxicity of different therapeutic schedules. As clearly shown in Fig 2B, no therapeutic schedules had any observable side effects at the low doses used in this study.

### Effect of different therapy schemes on DCE-MRI-derived parameters

**Fig 3:**
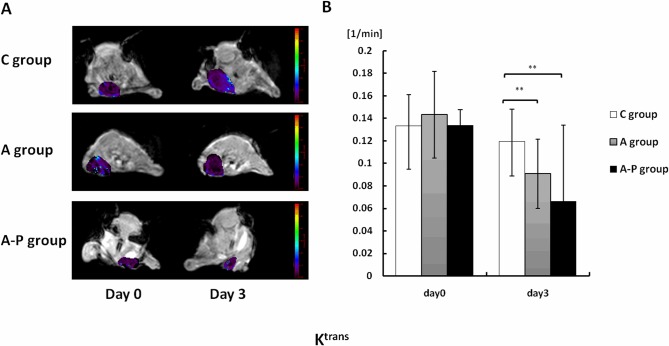
(A) Representative K^trans^ parametric maps of female athymic nude mice bearing orthotopic MDA-MB-435 tumors on days 0 and 3 after different treatments were initiated. Decreased K^trans^ values were observed on day 3 in both A and A-P groups. (B) Quantitative DCE-MRI region-of-interest analysis of K^trans^ values. **, P < 0.01.

**Fig 4:**
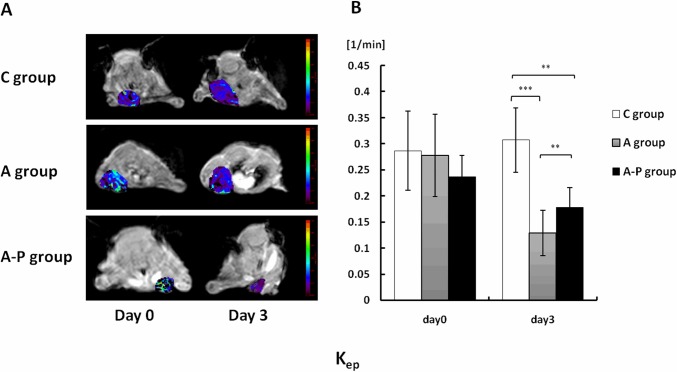
(A) Representative K_ep_ parametric maps of female athymic nude mice bearing orthotopic MDA-MB-435 tumors on days 0 and 3 after different treatments were initiated. Decreased K_ep_ values were observed on day 3 in both A and A-P groups. (B) Quantitative DCE-MRI region-of-interest analysis of K_ep_ values. **, P < 0.01; ***, P < 0.001.

**Fig 5:**
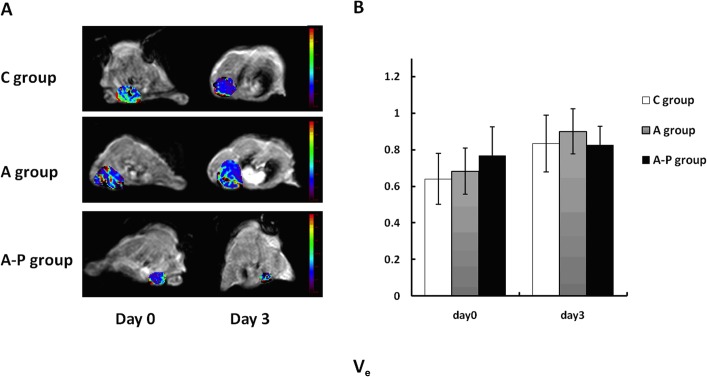
(A) Representative V_e_ parametric maps of female athymic nude mice bearing orthotopic MDA-MB-435 tumors on days 0 and 3 after different treatments were initiated. No significant change in V_e_ was found in A group and A-P group before and after treatment. (B) Quantitative DCE-MRI region-of-interest analysis of V_e_ values.

DCE-MRI data were acquired at day 0 (baseline, before treatment) and at day 3 after the treatment was started. Parametric maps of K^trans^, K_ep_ and V_e_ for all groups are presented in Figs 3A, 4A and 5A, respectively. Histograms of K^trans^, K_ep_ and V_e_ for each group are shown in Figs 3B, 4B and 5B, respectively. Little fluctuation of DCE-MRI-derived parameters was observed in the control mice on different days after tumor inoculation. Animals in the A and A-P groups showed significant decreases compared to those in the C group in terms of K^trans^ (Fig 3) at day 3. The average absolute K^trans^ values for these groups are listed in Table 1. The trend of K_ep_ values is similar to that of K^trans^ (Fig 4), and the average absolute K_ep_ values for these groups are listed in Table 2. The V_e_ value increased in all the treatment groups compared to the C group, but no significant difference was found between the A group and A-P group at the post-treatment time points examined (Fig 5). The average absolute V_e_ values for these groups are listed in Table 3.

**Table 1:** K^trans^ Values for Each Group.

Group	Baseline (/min)	Day 3 (/min)
C group	0.133±0.014	0.119±0.014
A group	0.143±0.019	0.090±0.015
A-P group	0.133±0.007	0.066±0.034

Values are presented as mean±SD.

**Table 2:** K_ep_ Values for Each Group.

Group	Baseline (/min)	Day 3 (/min)
C group	0.287±0.038	0.308±0.031
A group	0.278±0.039	0.129±0.022
A-P group	0.237±0.020	0.178±0.019

Values are presented as mean±SD.

**Table 3:** V_e_ Values for Each Group.

Group	Baseline	Day 3
C group	0.641±0.139	0.834±0.156
A group	0.683±0.126	0.900±0.123
A-P group	0.768±0.159	0.825±0.103

Values are presented as mean±SD.

### The inhibitory effect of Abraxane and its synergistic interaction with cisplatin on MDA-MB-435 tumor-induced proliferation

**Fig 6:**
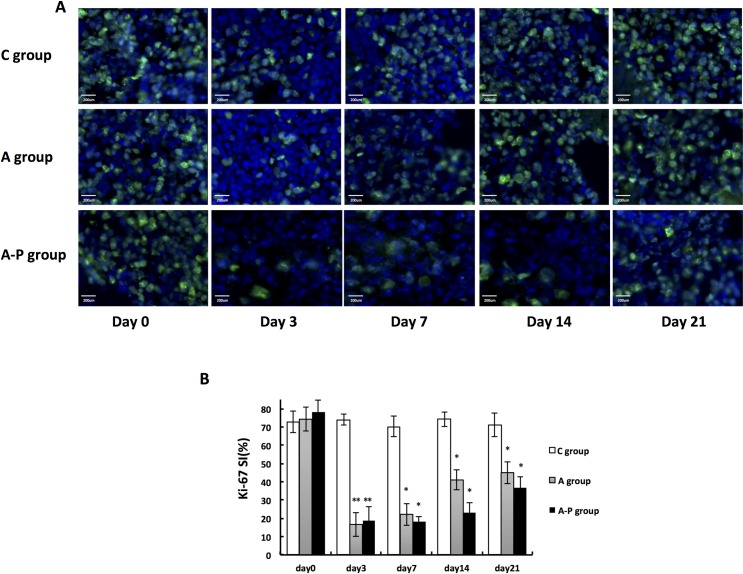
(A) Ex vivo Ki67 staining of tumor sections from PBS- and different treatment groups on days 0, 3, 7, 14, and 21. Green = Ki67; blue = DAPI. (B) Ki-67 SI calculated based on staining of MDA-MB-435 tumor sections from PBS- and different treatment groups on days 0, 3, 7, 14, and 21. *, P < 0.05; **, P < 0.01.

The immunofluorescence of Ki67 was used to quantify cell proliferation in MDA-MB-435 tumor sections from all groups to evaluate whether Abraxane in combination with cisplatin enhanced regression. In the C group animals, tumors contained a large fraction of Ki67-positive cells. The percentage of Ki67-positive cells in MDA-MB-435 tumors in the control mice receiving PBS was 73±6%, which was unchanged throughout the experiment (Fig 6). In contrast, a significant delay in cell proliferation was observed in all the treatment groups compared with the C group at all post-treatment time points.

### Morphology of tumor vascularity modulated by Abraxane in combination with cisplatin

**Fig 7:**
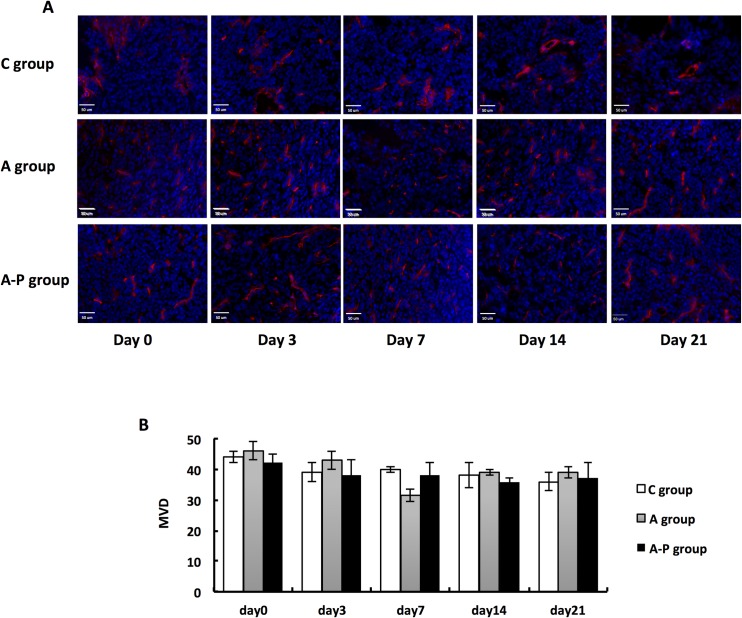
(A) Ex vivo CD31 staining of tumor sections from PBS- and different treatment groups on days 0, 3, 7, 14, and 21. Red = CD31; blue = DAPI. (B) Mean microvessel density calculated from CD31 staining.

**Fig 8:**
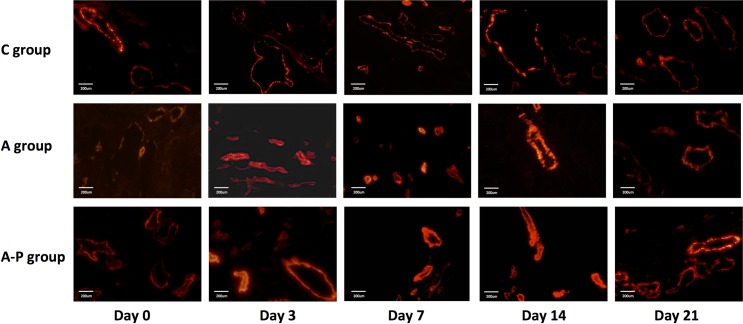
Ex vivo α-SMA staining of tumor sections from PBS- and different treatment groups on days 0, 3, 7, 14, and 21. Red = α-SMA

Strong CD31 staining was observed along the cell membranes of endothelial cells in all the tumor slices examined (Fig 7). There was a significant difference in morphology between the two treatment groups and the control group at different time points. The blood vessels in the control group were dilated and irregular, whereas those in the treated group tended to be small and regular. Based on CD31 immunofluorescence staining, we also quantified MVD in treated and control tumors. As shown in Fig 8, there was no obvious difference in MVD among the C group, A group and A-P group at different time points.

To understand what causes vascular remodeling and changes in vascular permeability during and after therapy at the molecular level, smooth muscle actin (α-SMA) expression was evaluated with immunofluorescence staining (Fig 8). The stains with α-SMA revealed a difference in the distribution of vascular smooth muscle cells in the three groups. Fig 8 shows that the vessels in the C group are leakier, more dilated, more tortuous and have fewer vascular smooth muscle cells. In contrast, vessels of all the treatment groups show more prominent α-SMA immunoreactivity and greater coverage by vascular smooth muscle cells.

## Discussion

Several clinical trials documented that Abraxane had improved efficacy and favorable safety in the treatment of recurrent or metastatic disease [3, 4]. It has also been shown to play an important role in inhibiting the migration of endothelial cells to anti-angiogenic cells both *in vitro* and *in vivo* [7]. However, the understanding of the pathophysiology of tumor vascularization and its change during anti-cancer therapy is incomplete. The current approach to quantifying tumor vasculature is mainly based on immunohistochemistry. The inherent anatomical and physiological heterogeneity of tumors, potential variability in the measurement method, multiple samples required at any given time, ethical and physical limitations [18], and other factors, are the main drawbacks that severely affect immunohistochemistry results and hinder the interpretation of the observed therapeutic effects.

The ability to non-invasively visualize and quantify tumor vasculature *in vivo* at a functional level will provide a new opportunity to understand the mechanism of angiogenesis, anti-tumor blood vascular treatment and treatment efficacy. Non-invasive imaging modalities include MRI, PET, SPECT, CT and optical imaging are rapidly emerging fields in investigating the structure and function of tumor microvasculature and microcirculation [15–17]. MRI gives high special resolution, good soft tissue contrast and it can provide both anatomical and functional information, especially with the use of DCE-MRI technique, for quantitative and accurate analysis of tumor angiogenesis in real time[15,16]. In this study, we investigated the potential of DCE-MRI to accurately assess the vascular response of human mammary carcinomas to Abraxane treatment and the effectiveness of its synergistic interaction with cisplatin. Treatment with Abraxane in combination with cisplatin influenced the vascular characteristics of MDA-MB-435 xenografts, as shown by a significant decrease in K^trans^ and K_ep_ at day 3. Moreover, this reduction in K^trans^ and K_ep_ was not completely correlated with the caliper-measured tumor response. Significant inhibition of tumor growth was not observed until day 7 after therapy, which is later than can be detected by DCE-MRI. This finding indicates that DCE-MRI is able to detect early responses preceding clinical regression.

To confirm that variations in K^trans^ and K_ep_ reflect changes in vessel flow and permeability, immunohistochemical staining of CD31 and α-SMA was performed. Histological analysis of tumor tissue showed a significant change in the morphology of tumor vessels in both treatment groups. The diameter of the blood vessels in the PBS-treated group was larger, whereas that in all treated groups tended to be smaller. No significant difference in vessel density was observed in the treatment groups. More importantly, a significantly enhanced immunoreactivity of α-SMA and greater coverage by vascular smooth muscle cells on vessels were observed in the A and A-P groups over the PBS control group. This result suggests that K^trans^ and K_ep_ are sensitive enough to detect the changes in microvasculature 3 days after treatment; these morphological and functional alterations are mainly induced by Abraxane and appear to reflect vasculature normalization induced by the treatment [14].

The tumor vessel normalization and maturation caused by Abraxane improve the efficiency of cisplatin delivery. These improved conditions explain why continuous tumor growth suppression was observed in the A-P group, whereas tumor regrowth was found in the A group after the treatment was halted. Our observations are in accordance with previous findings [14, 19, 20]. These studies show that one of the indirect effects of taxanes is the inhibition of the expression of pro-angiogenic factors and VEGF. These effects may result in a remodeling of the cancer vasculature, improved conditions in the tumor, further treatment benefits, and reducing the progress of malignancy [14, 19, 20].

In DCE-MRI, the V_e_ value is the extravascular extracellular space (EES) volume fraction, and a proliferative index reduction will eventually lead to an increase in the size of the EES [15, 16]. Histological analysis showed a decreased percentage of Ki67-positive nuclei at days 3, 7 and 14 in all treated groups compared to the control. Although a slight increase in V_e_ values was found in treated groups at day 3, the change was statistically significant. Thus, V_e_ does not seem to be sensitive enough to detect the changes in the size of the extravascular extracellular space in this study.

However, There are limitations of this study. DCE-MR imaging of MDA-MB-435 tumor mice was conducted with a clinical 3.0 T system, not a small animal MR. Not only that, the tumor size was significantly reduced after given different therapeutic schedules in all treatment groups. Some tumor samples may be too small to reveal differences that may exist between the various therapeutic schedules during the DCE-MRI analysis and this finding could lead to a statistic bias in a small sample study [17, 21]. Therefore, high spatiotemporal resolution DCE-MRI protocol for clinical settings may need further development.

## Conclusion

Our data illustrate that the variation in permeability in the vasculature induced by Abraxane could be quantitatively visualized by DCE-MRI, demonstrating this to be a useful method to study early treatment response in breast cancer. K^trans^ and K_ep_ values are promising MR imaging biomarkers that allow for the noninvasive evaluation of the vascular response to treatment before size changes can be found. These markers also have the potential to provide opportunities to adjust anticancer drug doses and intervals early enough to maintain a sustained anti-tumor effect and avoid relapse. Collectively, the histological results also support the mechanisms of action of Abraxane on tumor vasculature based on a change in morphological, functional and molecular characteristics, inducing transient "normalization" of the abnormal structure. Vascular remodeling by Abraxane improves the efficiency of cisplatin delivery, and the combination therapy is more effective than Abraxane alone.
