# Correction: DCE-MRI-Derived Parameters in Evaluating Abraxane-Induced Early Vascular Response and the Effectiveness of Its Synergistic Interaction with Cisplatin

**DOI:** 10.1371/journal.pone.0290861

**Published:** 2023-08-25

**Authors:** Xilin Sun, Lili Yang, Xuefeng Yan, Yingying Sun, Dongliang Zhao, Yang Ji, Kai Wang, Xiaoyuan Chen, Baozhong Shen

This notice corrects the following errors in the Results section of [1]:

- The wrong image data were reported in Fig 6A and used to generate the quantitative results in Fig 6B. Replacement image data from the original experiment and updated quantification results have been provided in the updated Fig 6 with this notice. The results statement referring to these data is hereby updated to report the correct percentage value: “The percentage of Ki67-positive cells in MDA-MB-435 tumors in the control mice receiving PBS was 55.67±3%, which was unchanged throughout the experiment (Fig 6).”
- In Fig 7A, the A group Day 0 image was duplicated in the Day 3 and Day 14 results, and the A-P Day 0 image was duplicated in the A group Day 21 panel. An updated version of Fig 7 is provided here in which all panels have been replaced with data from a replicate experiment. Mean microvessel density data were reanalyzed using the correct image data, and the results of the reanalysis are reported in the updated Fig 7B.
- The wrong figure was cited in the fifth sentence of the first paragraph of the “Morphology of tumor vascularity modulated by Abraxane in combination with cisplatin” subsection. The sentence is corrected to: “As shown in Fig 7, there was no obvious difference in MVD among the C group, A group and A-P group at different time points.”

**Fig 6:**
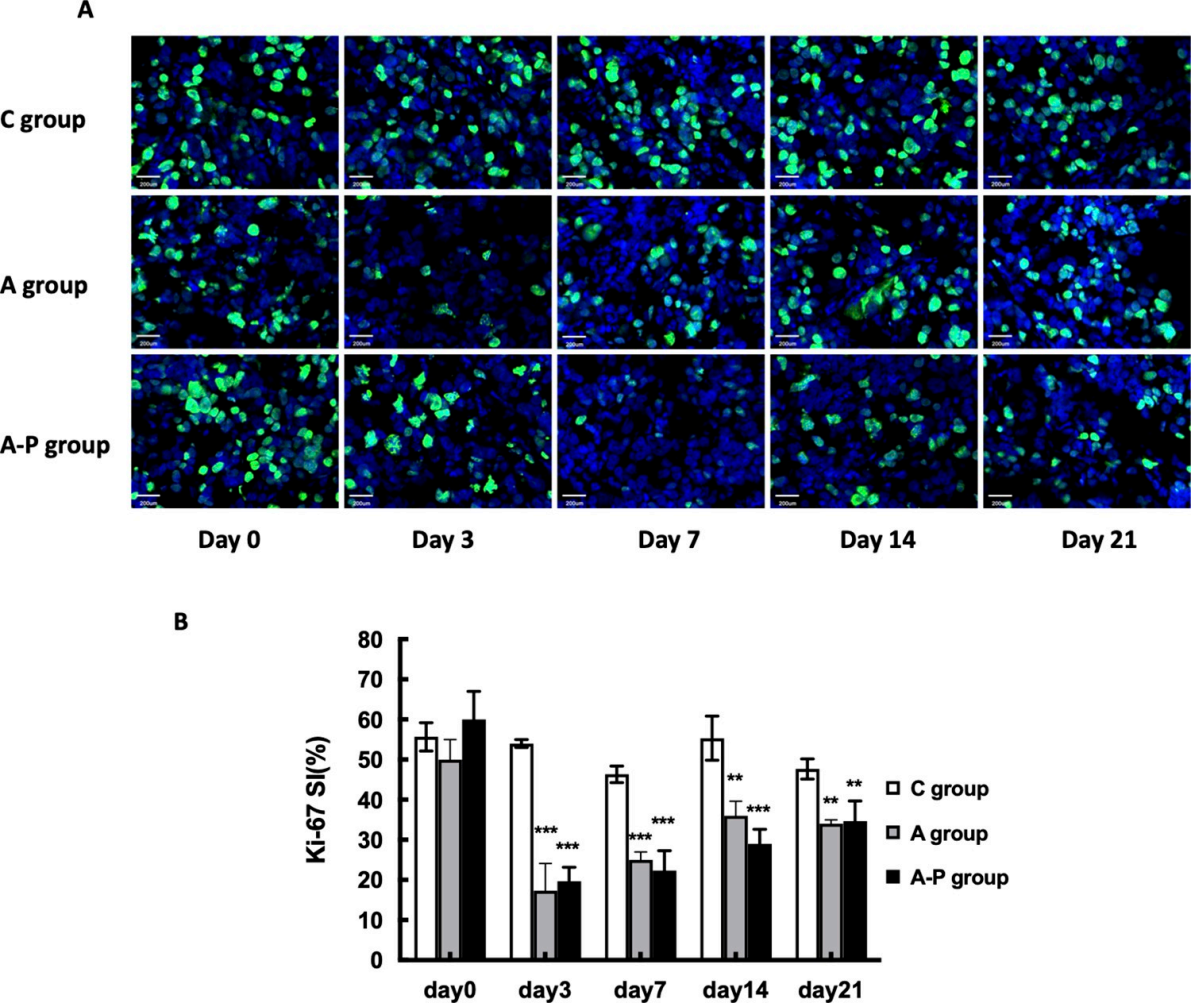
(A) Ex vivo Ki67 staining of tumor sections from PBS- and different treatment groups on days 0, 3, 7, 14, and 21. Green = Ki67; blue = DAPI. (B) Ki-67 SI calculated based on staining of MDA-MB-435 tumor sections from PBS- and different treatment groups on days 0, 3, 7, 14, and 21. *, P < 0.05; **, P < 0.01.***, P < 0.001.

**Fig 7:**
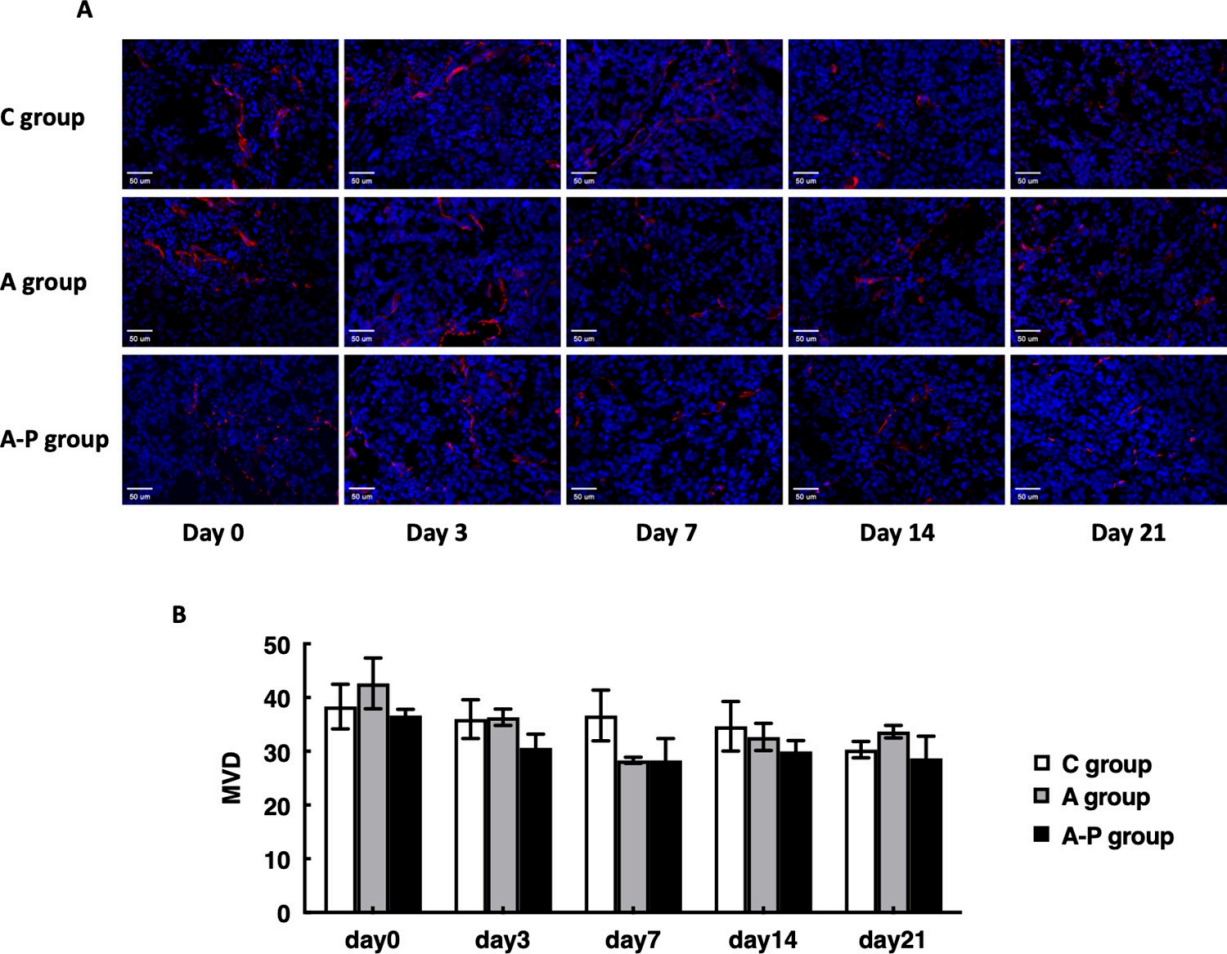
(A) Ex vivo CD31 staining of tumor sections from PBS- and different treatment groups on days 0, 3, 7, 14, and 21. Red = CD31; blue = DAPI. (B) Mean microvessel density calculated from CD31 staining.

The underlying data supporting the updated versions of Figs 6 and 7 are provided in S1–S3 Files with this notice.

The corrected figures have been reviewed by a member of the *PLOS ONE* editorial board. They commented that the selected images from the initial publication and the updated Fig 6 do not align with the quantitative graphs. The authors confirmed that the data presented in the new panels do not align with the originally published graphs, but clarified that although there are differences between the original published graph and the updated graph in the corrected figure, the overall trend remains and the conclusions are not affected.

The editorial board member also stated that the figures clearly show that combination therapy is very effective in reducing tumor proliferation rate, as a potentially effective treatment, but that the data and histology do not show modified vascularization as stated in the conclusions. Furthermore, the board members stated that drug synergism outcomes, as suggested in the title, is also not discernible due to the lack of Cis only cohort. Furthermore, they commented that the conclusion that Abx improves Cis delivery is not clearly defined without pharmacokinetic assessment of drug in tumor tissues. The authors’ response confirmed that there was no obvious difference in MVD among the C group, A group, and A-P groups, but indicated that the CD31 immunofluorescence staining results presented blood vessels in the control group that were dilated and irregular, whereas those in the treated groups tended to be small and regular. The authors also pointed out that the α-SMA staining results presented in Fig 8 supported the conclusions. The authors clarify that the definitions of dilated and irregular differs per individual and that this should be acknowledged as a limitation to the study.

As part of this review, the authors have provided the following additional information to clarify the methodology of the reported study:

- The MDA-MB-435 tumor model was established via the injection of 8×10^6^ cells in the left mammary fat pad of athymic nude mice (female, 6 weeks). The mice were used for studies when the tumor volume reached about 250 +/-60 mm^3^ (14~21 d after implantation). Then, the tumor-bearing mice were randomly divided into three groups (n = 10/group) and underwent baseline DCE-MRI (day 0).
- Treatment, including PBS for the control group, the second Abraxane for A group and the second cisplatin for A-P group, was administered at day 3. These treatments were administered in the morning and the DCE-MR imaging was performed as late as possible to allow time for drug distribution and treatment. The S1 Table below presents a schematic of the experimental design for the DCE-MR imaging of treatment efficacy and ex vivo histopathology.
- In the methodology section for the ROI measurement, the authors referred to previous studies which used Philips Healthcare software. The current study was pre-clinical and different from the referred study; the current study required calculation of the arterial input function (AIF) first and the ROI covered the large vessels. The aorta cross sectional area was very small for the Axial MR mice imaging, so the ROI size was smaller. During assessment of the tumors, effort was made to try and use a consistent size for the ROI and the aorta. Tumor ROI Measurements were repeated 3~5 times and the liquefacient necrotic areas were excluded. The sizes of ROIs and the T10 values were also recorded by the software for calculating the resulting output maps, including K^trans^, V_e_, and K_ep_.
- The “Statistical Analysis” section of the Methodology should be updated to: “All quantitative data were expressed as means ± SD. Statistical comparisons of sequence-dependent effects of each time-point were conducted by one way ANOVA, followed by the Dunnett’s multiple comparisons test, was performed (GraphPad Prism 8.0.2). P values <0.05 were considered statistically significant.”

The article’s [1] Data Availability Statement provides links for the publicly available DCE-MRI DICOM data, but the yunpan.cn domain is no longer publicly available. The authors indicate that these data remain available upon request, and the Data Availability Statement of this article now should read: All original data underlying the results reported in this article are available upon request from the corresponding author.

The corresponding author apologizes for the errors in the article.

## Supporting information

S1 File Underlying image data Fig 6. (ZIP)

S2 File Underlying image data Fig 7. (ZIP)

S3 File Individual level data underlying the Figs 6 and 7 graphs. (ZIP)

S1 Table Schematic representing experimental design for DCE-MR imaging of treatment efficacy and ex vivo histopathology (DOCX)
