# Supplementary figures and images for: Correction: DCE-MRI-Derived Parameters in Evaluating Abraxane-Induced Early Vascular Response and the Effectiveness of Its Synergistic Interaction with Cisplatin
Source: PLoS One. 2023 Aug 25;18(8):e0290861. doi: 10.1371/journal.pone.0290861 (PMC10456182; doi:10.1371/journal.pone.0290861)

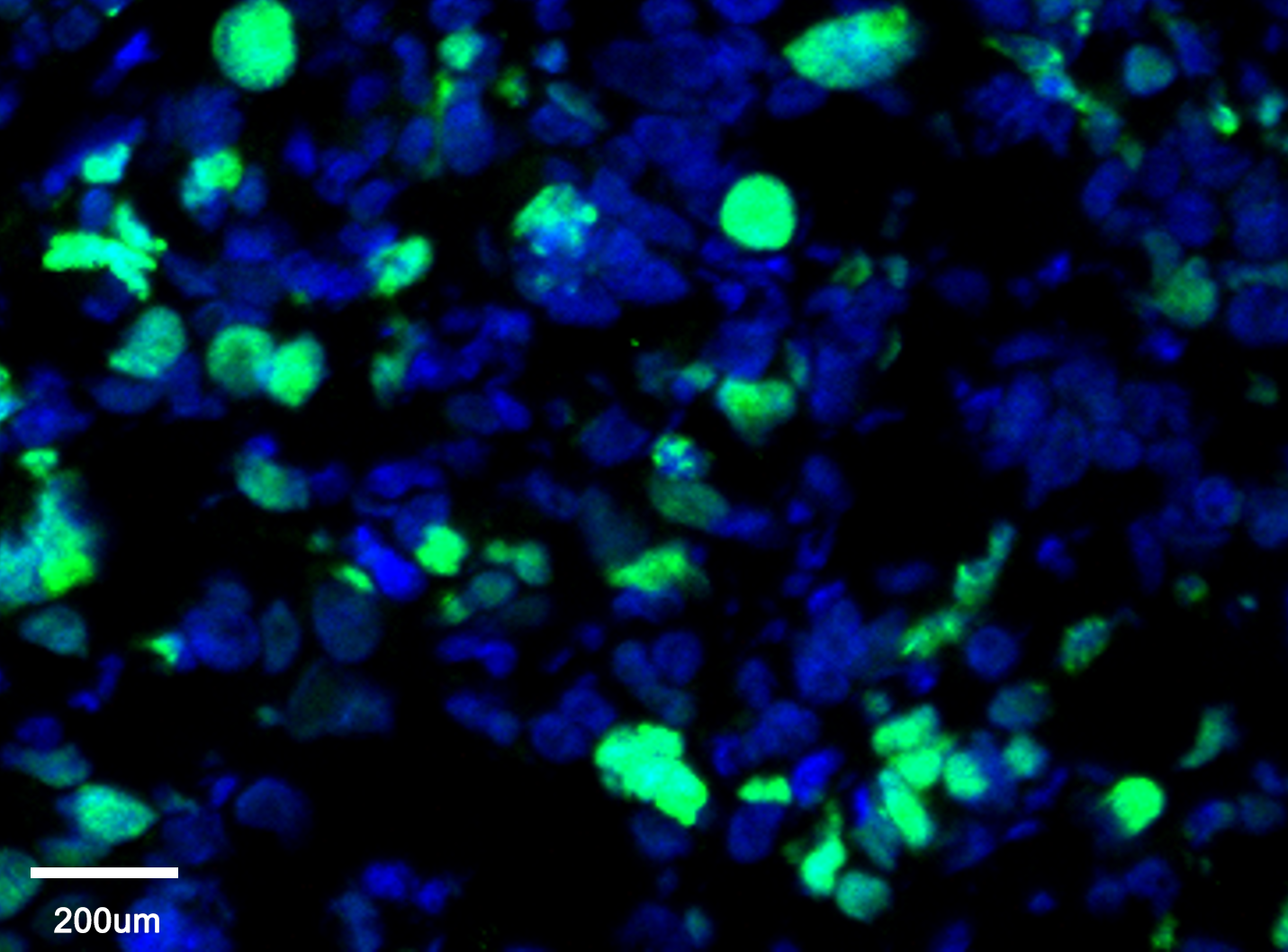

Supplement: S1 File — (ZIP) [file pone.0290861.s001.zip › S1 File/A group_Day0.tif]

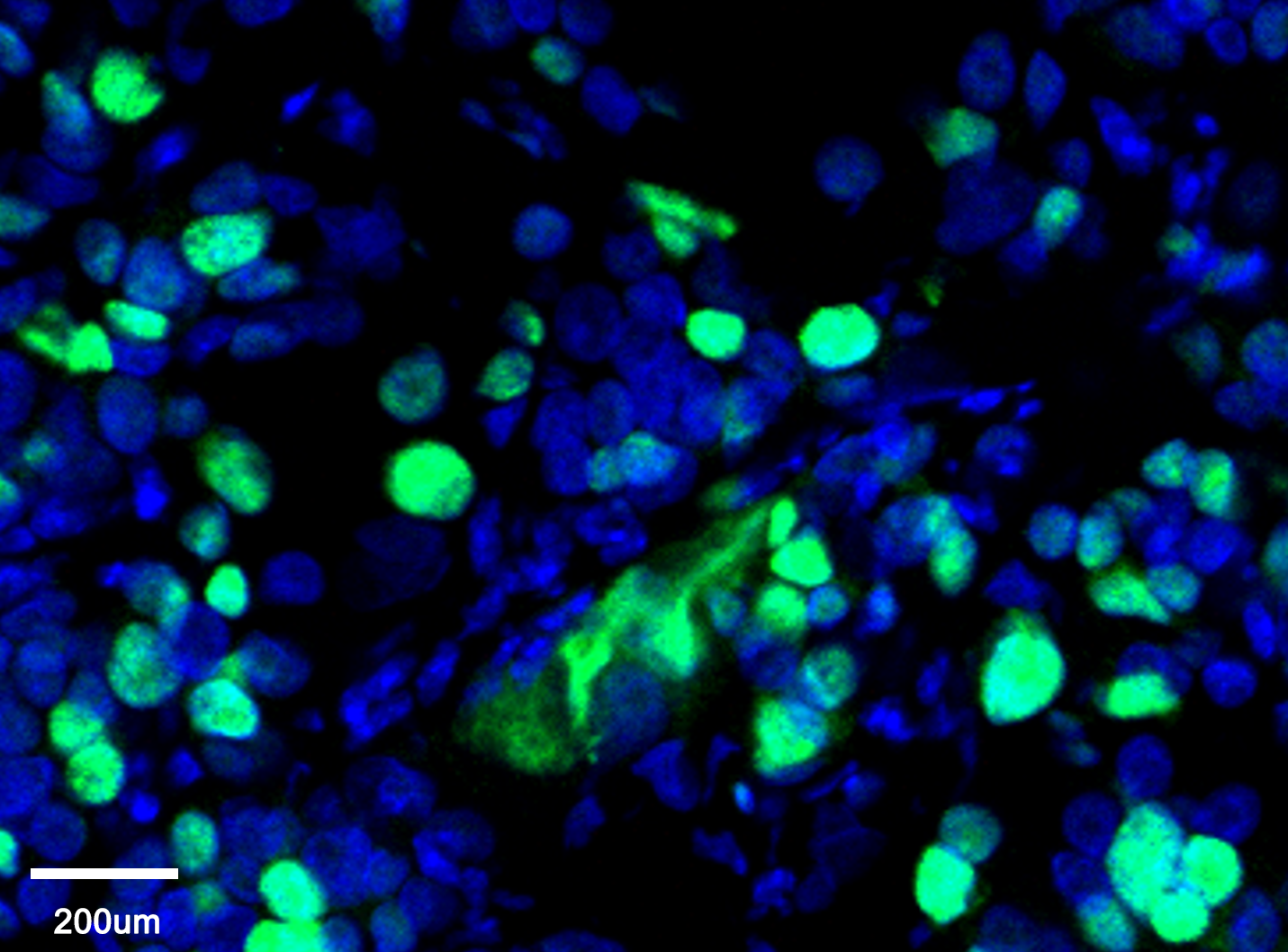

Supplement: S1 File — (ZIP) [file pone.0290861.s001.zip › S1 File/A group_Day14.tif]

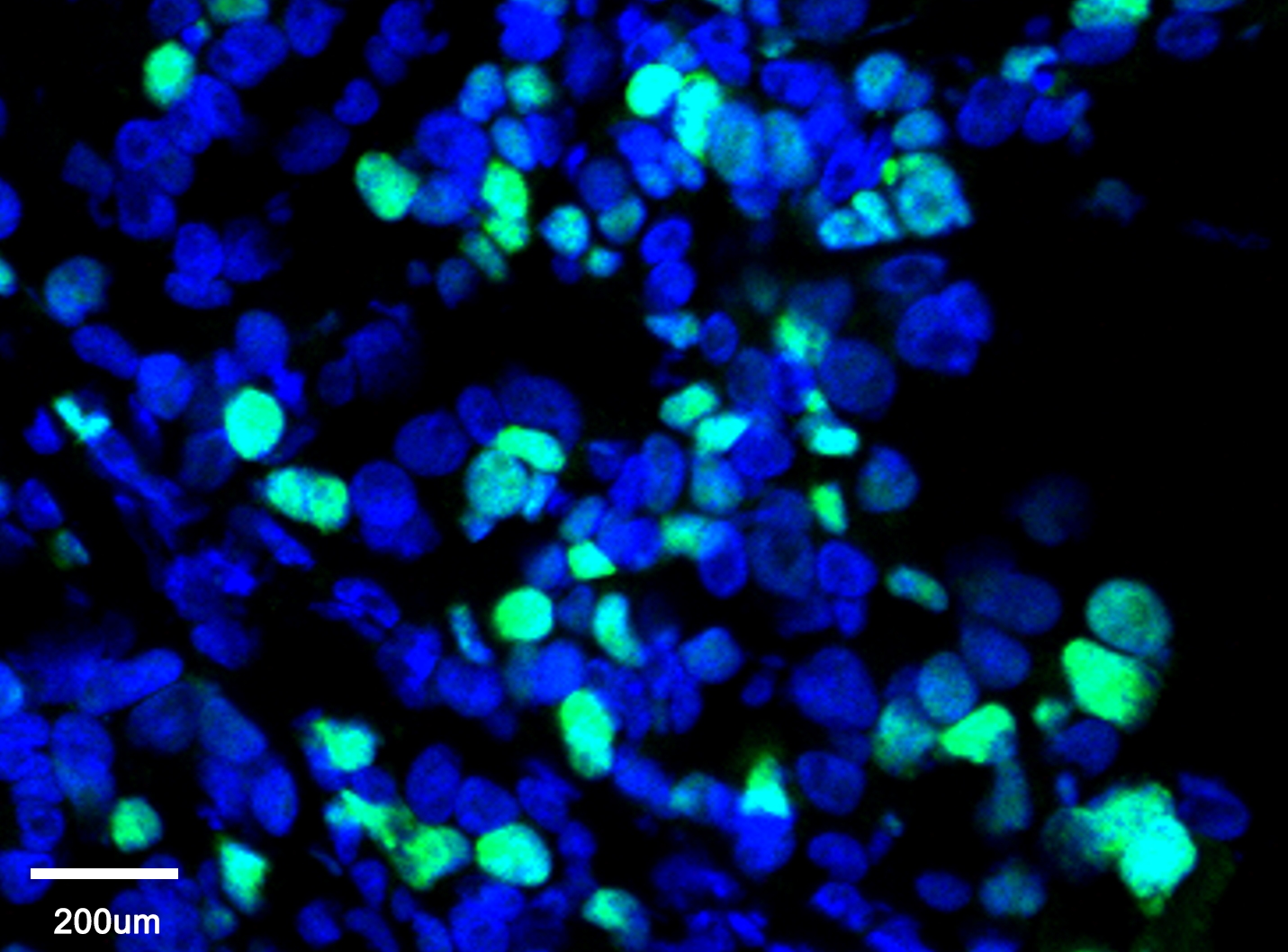

Supplement: S1 File — (ZIP) [file pone.0290861.s001.zip › S1 File/A group_Day21.tif]

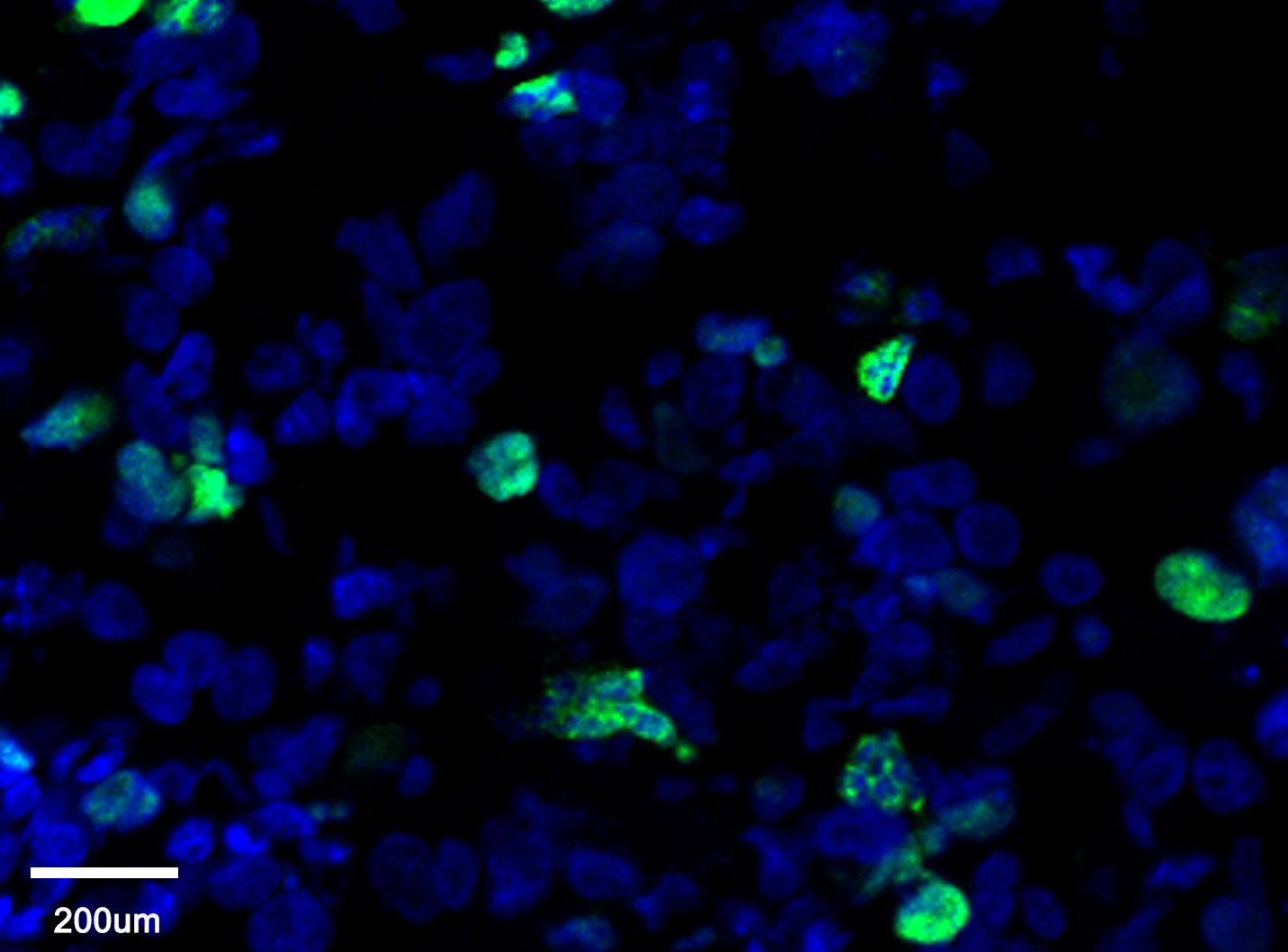

Supplement: S1 File — (ZIP) [file pone.0290861.s001.zip › S1 File/A group_Day3.tif]

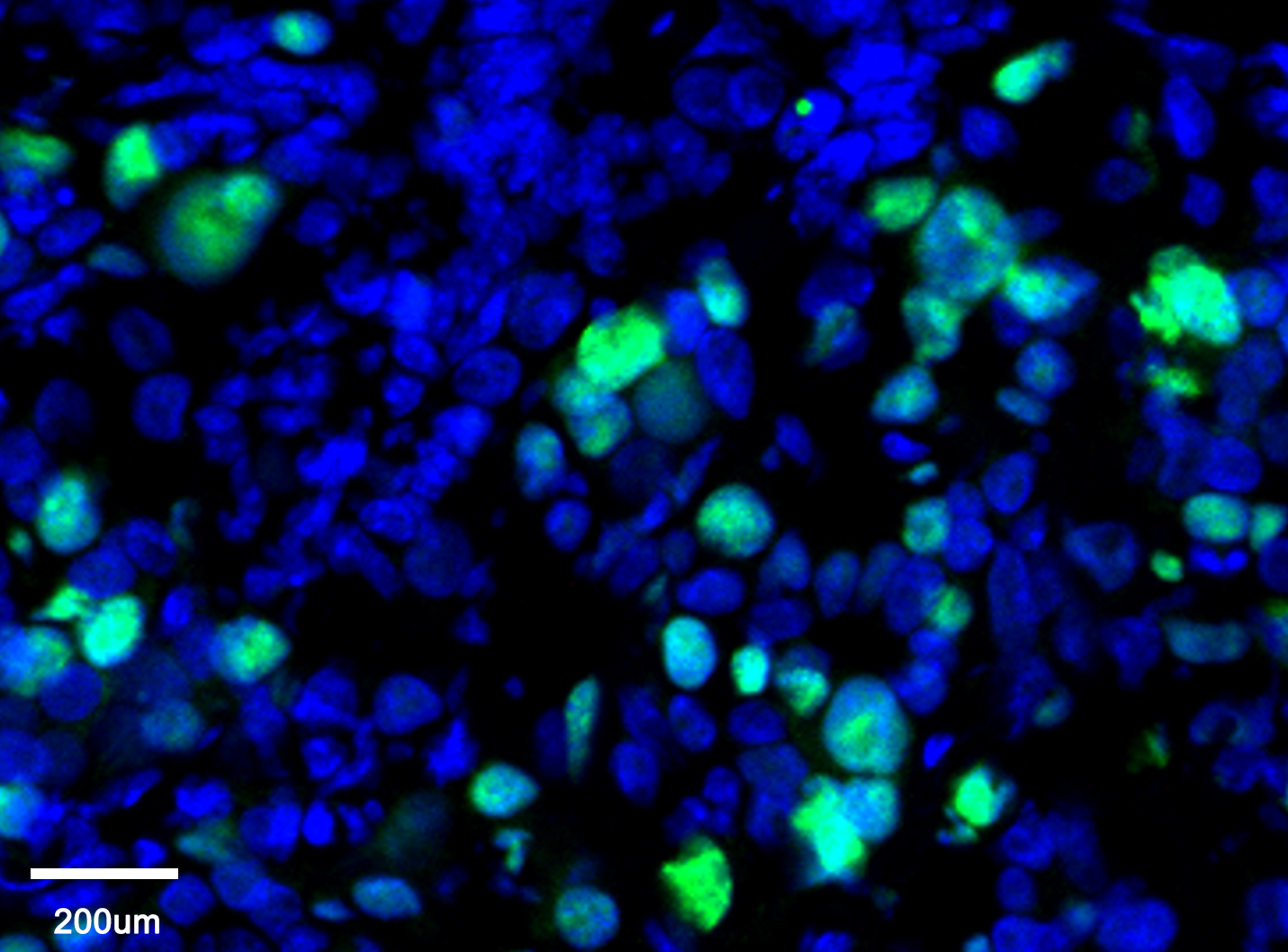

Supplement: S1 File — (ZIP) [file pone.0290861.s001.zip › S1 File/A group_Day7.tif]

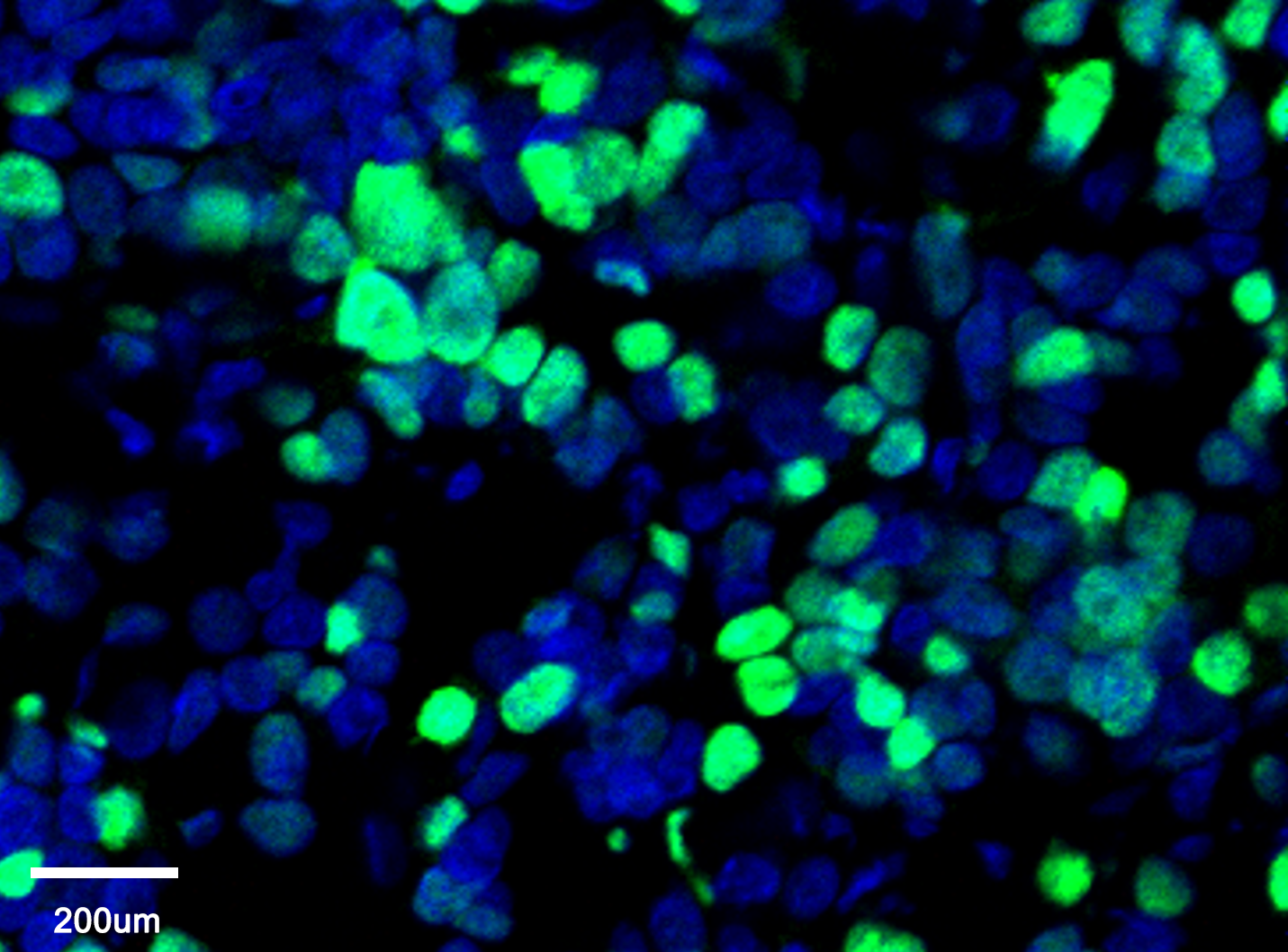

Supplement: S1 File — (ZIP) [file pone.0290861.s001.zip › S1 File/A-P group_Day0.tif]

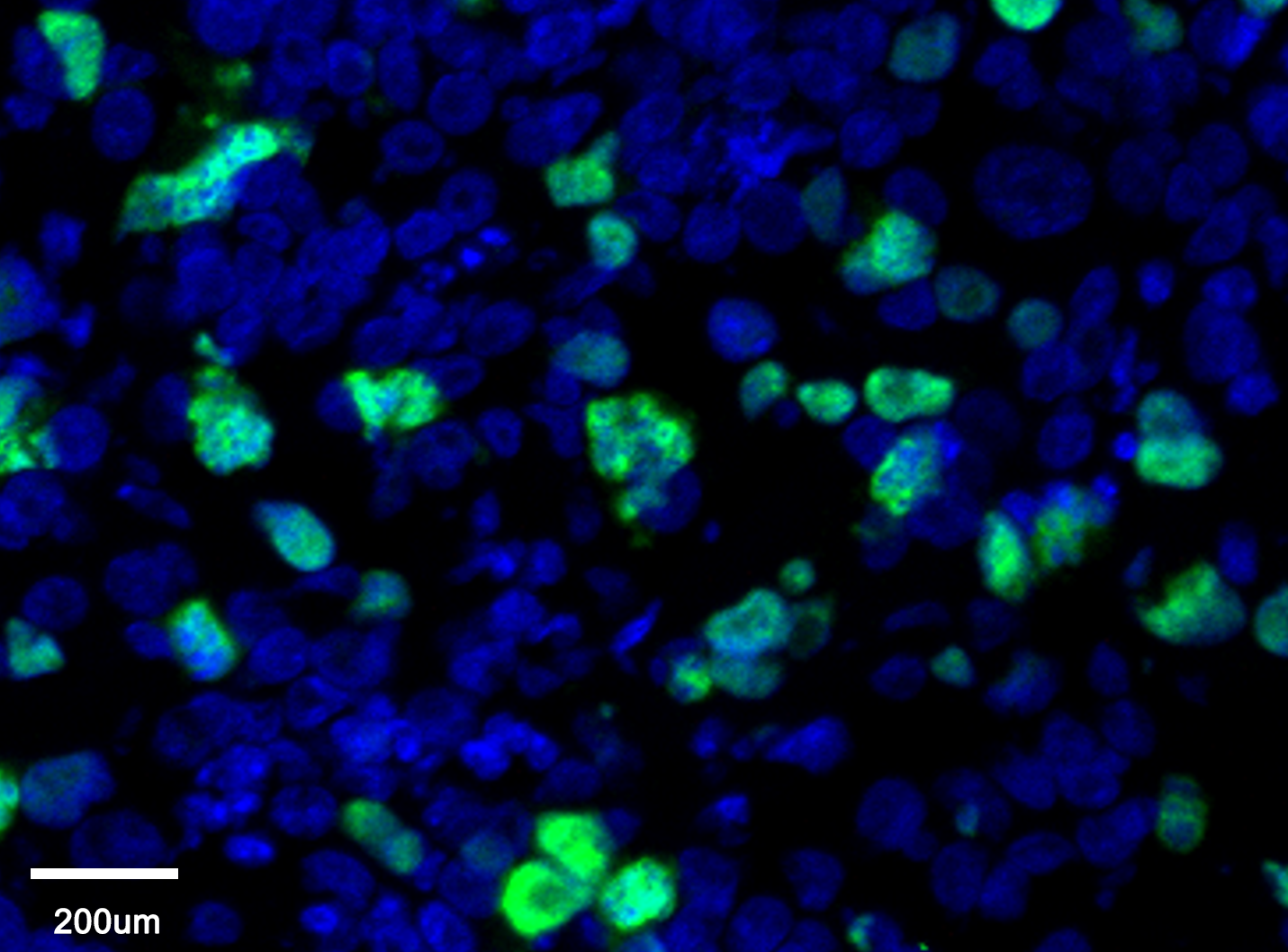

Supplement: S1 File — (ZIP) [file pone.0290861.s001.zip › S1 File/A-P group_Day14.tif]

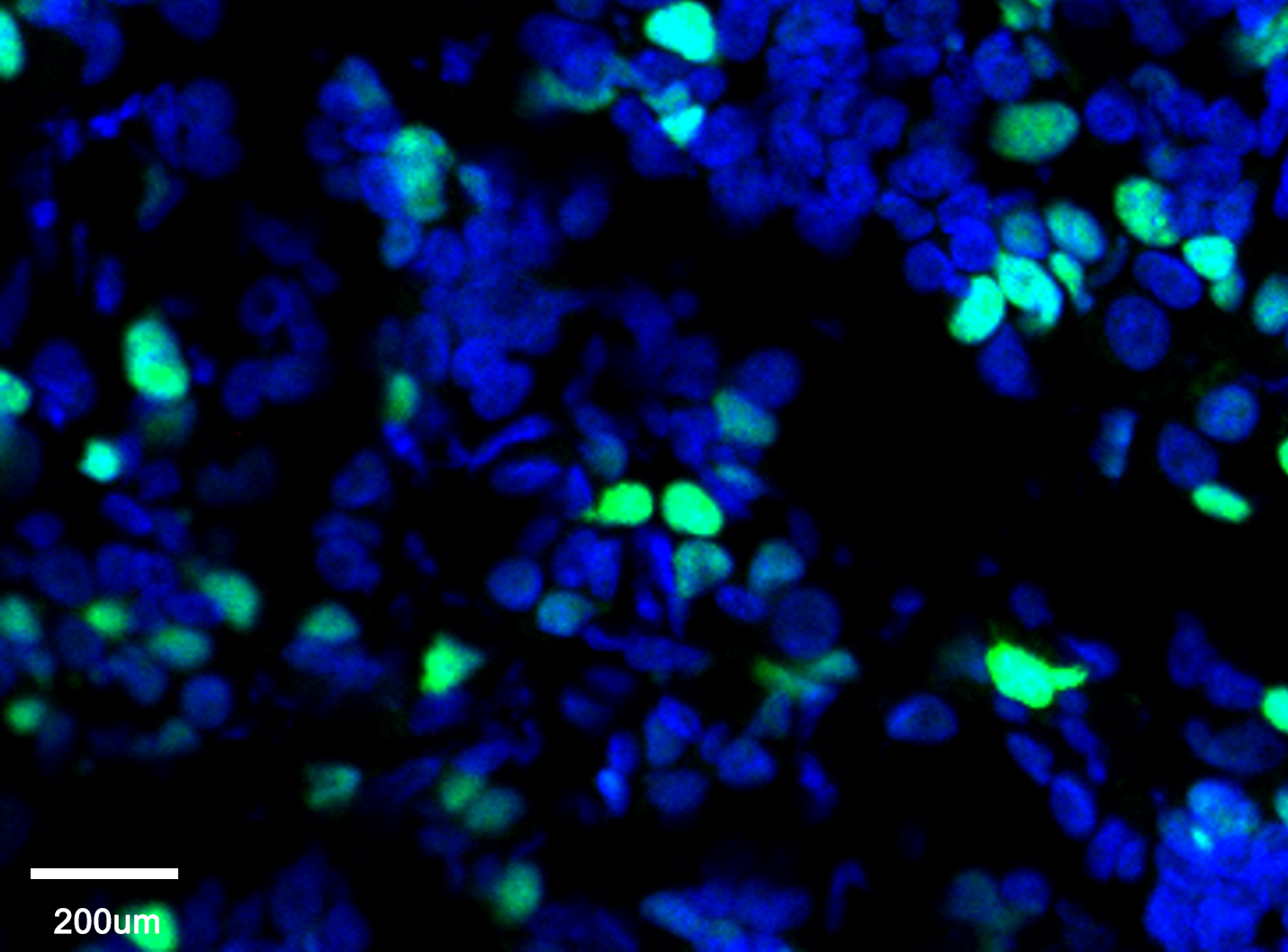

Supplement: S1 File — (ZIP) [file pone.0290861.s001.zip › S1 File/A-P group_Day21.tif]

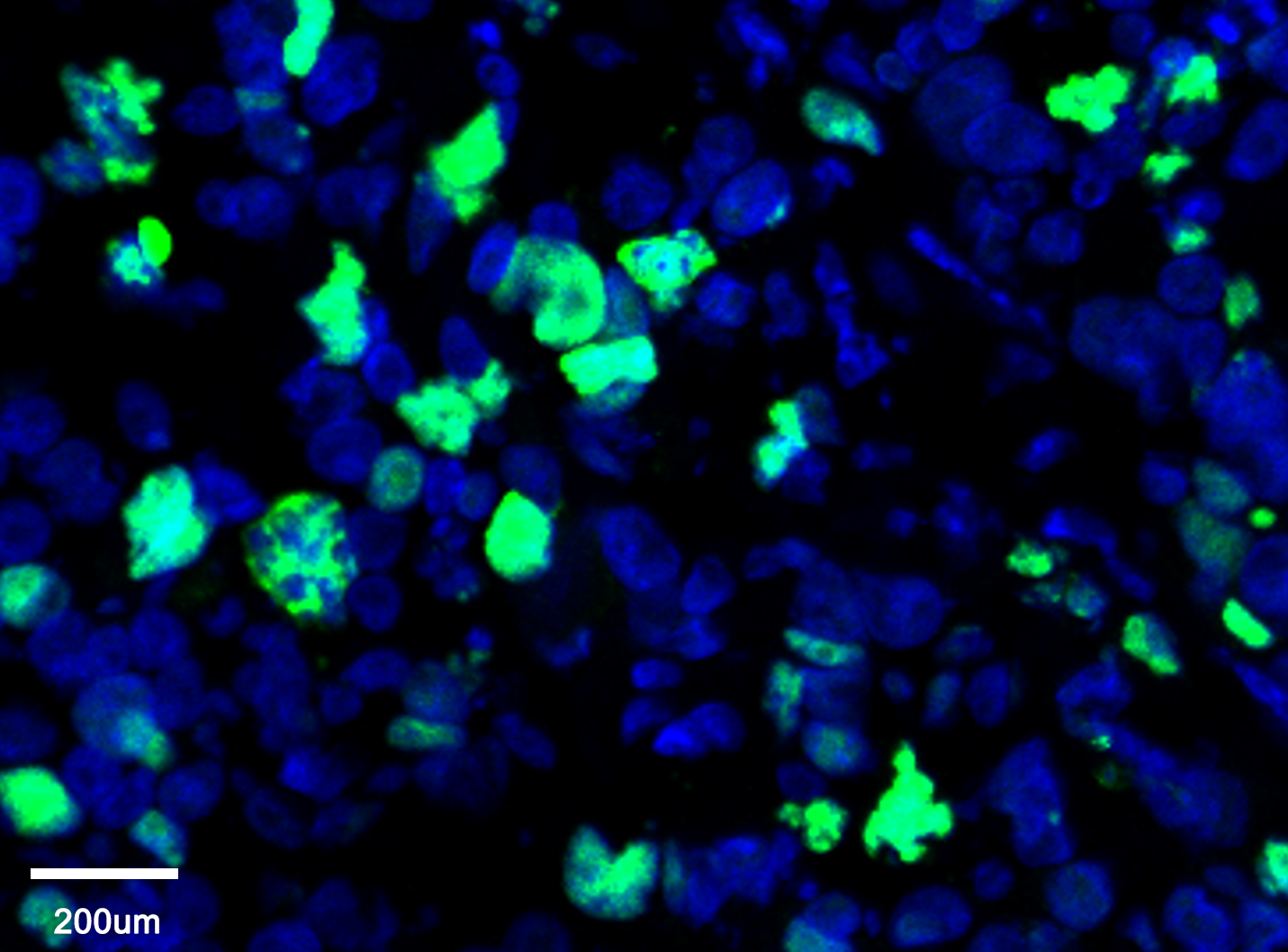

Supplement: S1 File — (ZIP) [file pone.0290861.s001.zip › S1 File/A-P group_Day3.tif]

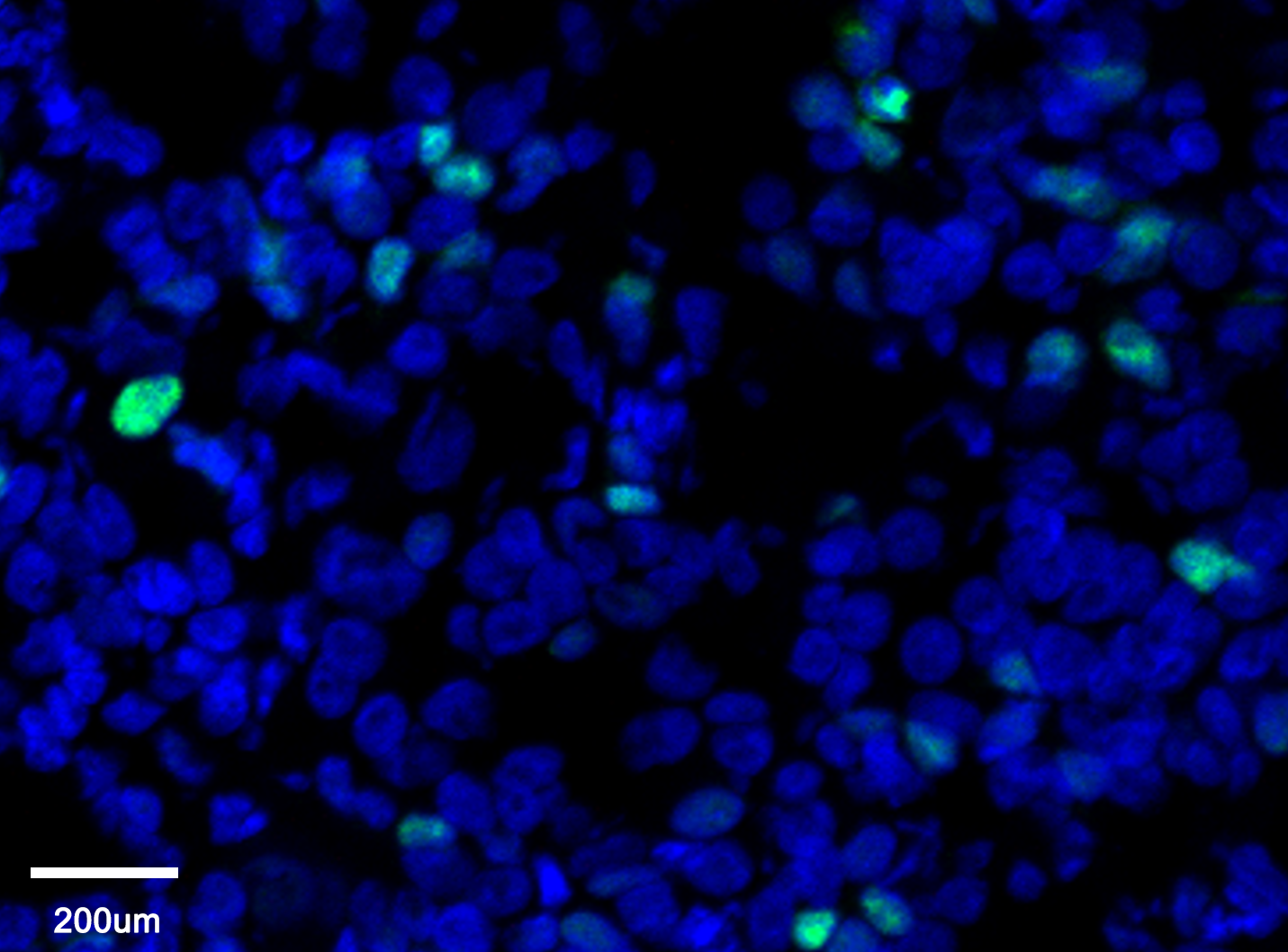

Supplement: S1 File — (ZIP) [file pone.0290861.s001.zip › S1 File/A-P group_Day7.tif]

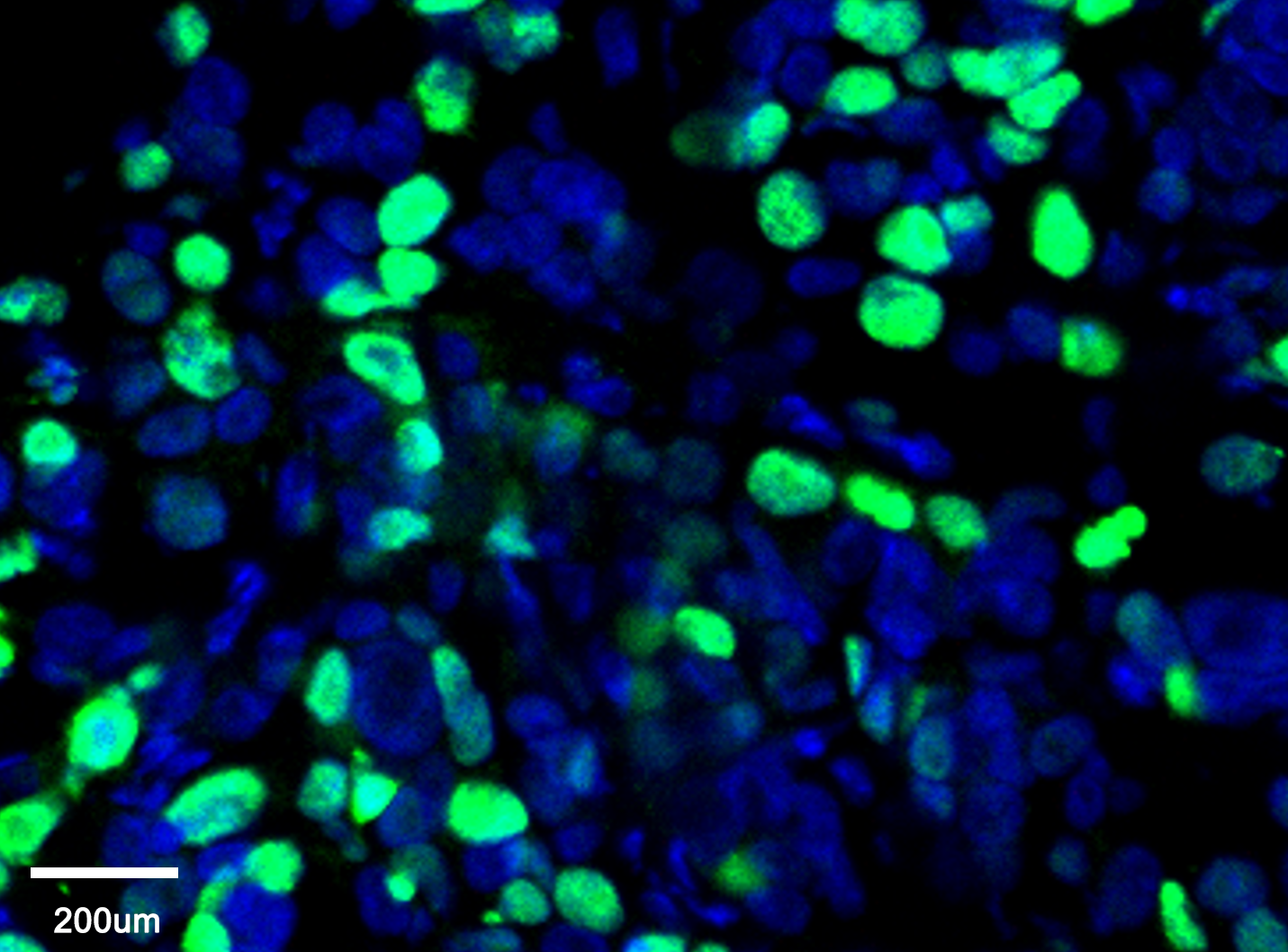

Supplement: S1 File — (ZIP) [file pone.0290861.s001.zip › S1 File/C group_Day0.tif]

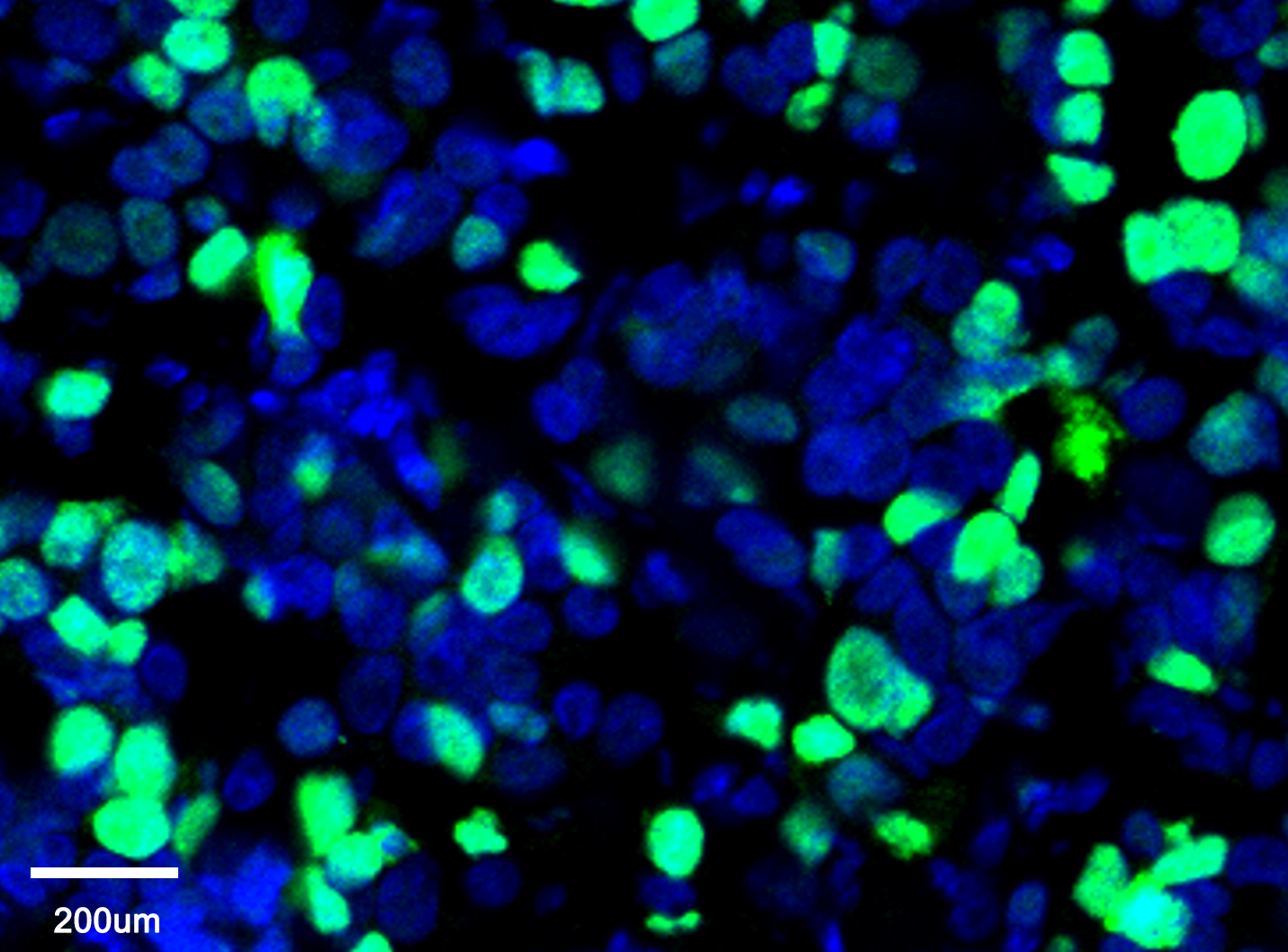

Supplement: S1 File — (ZIP) [file pone.0290861.s001.zip › S1 File/C group_Day14.tif]

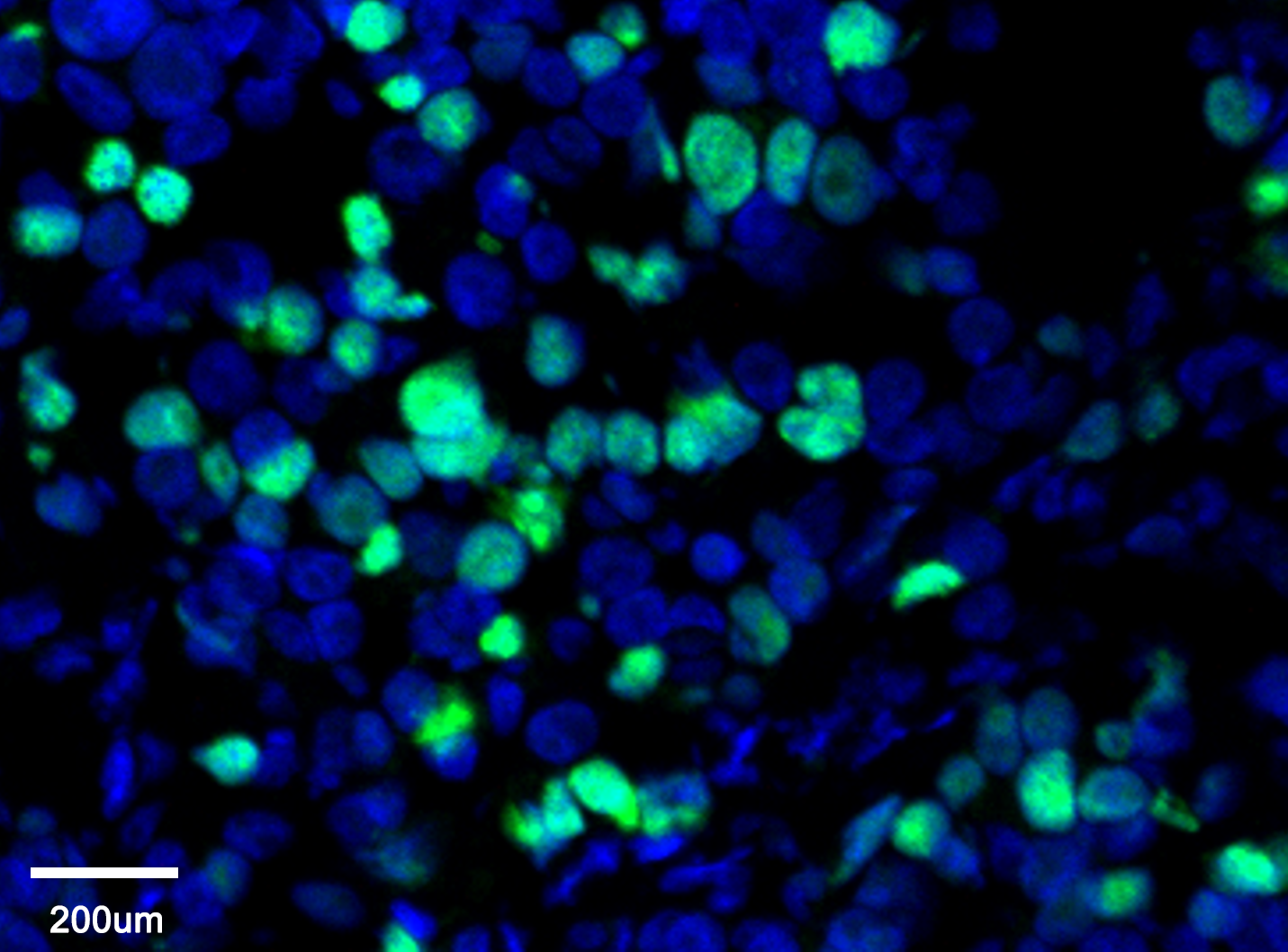

Supplement: S1 File — (ZIP) [file pone.0290861.s001.zip › S1 File/C group_Day21.tif]

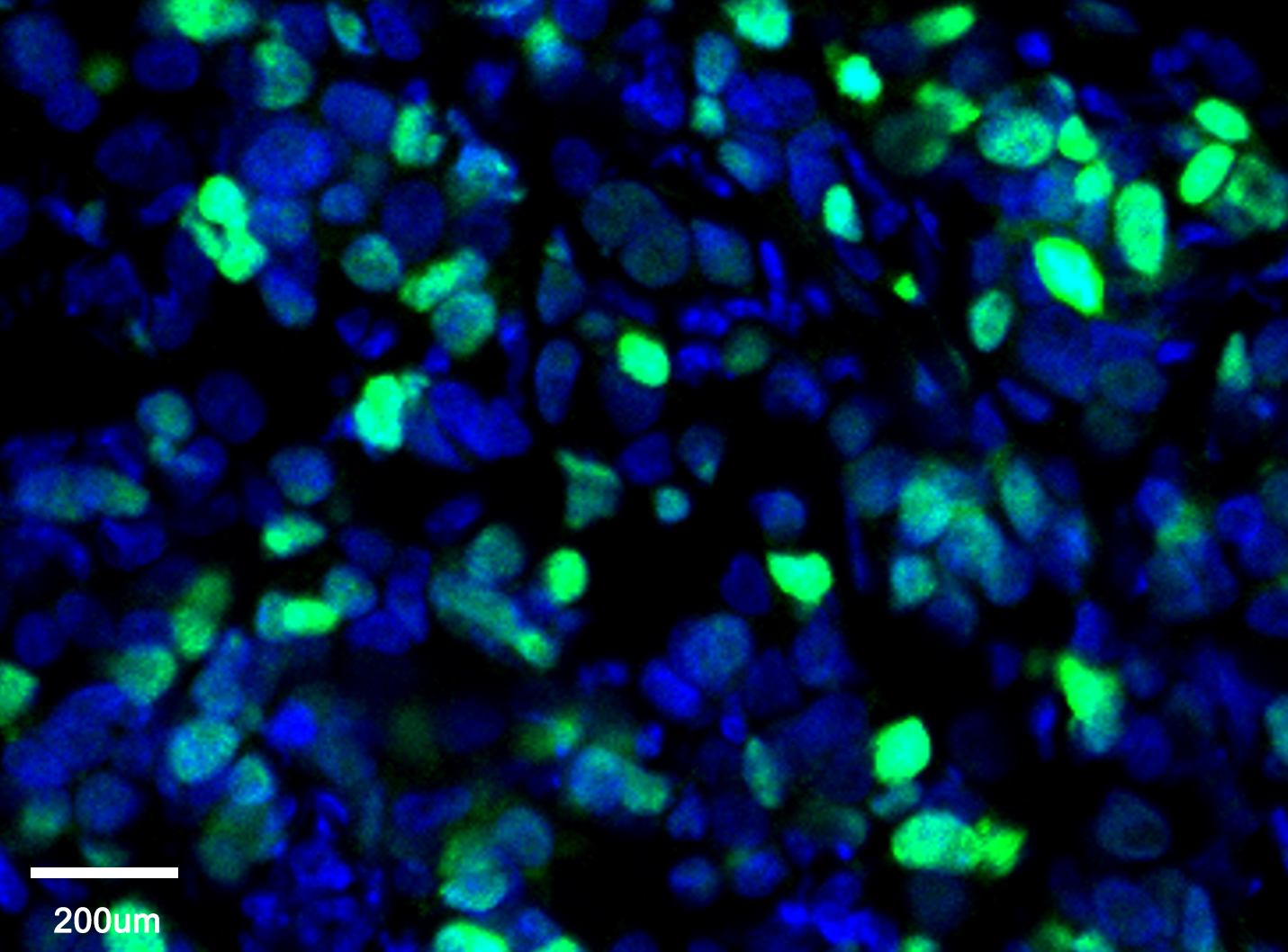

Supplement: S1 File — (ZIP) [file pone.0290861.s001.zip › S1 File/C group_Day3.tif]

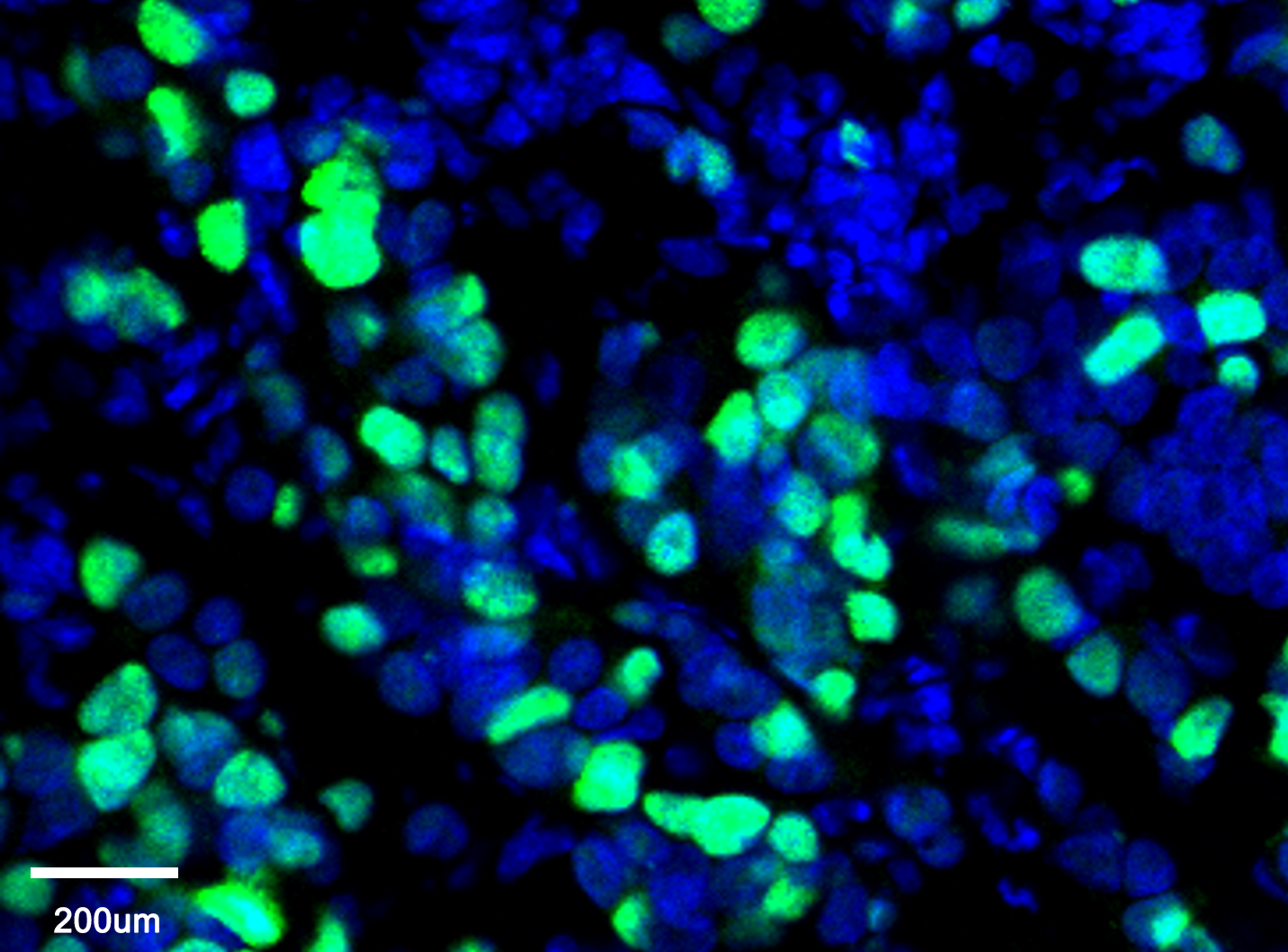

Supplement: S1 File — (ZIP) [file pone.0290861.s001.zip › S1 File/C group_Day7.tif]

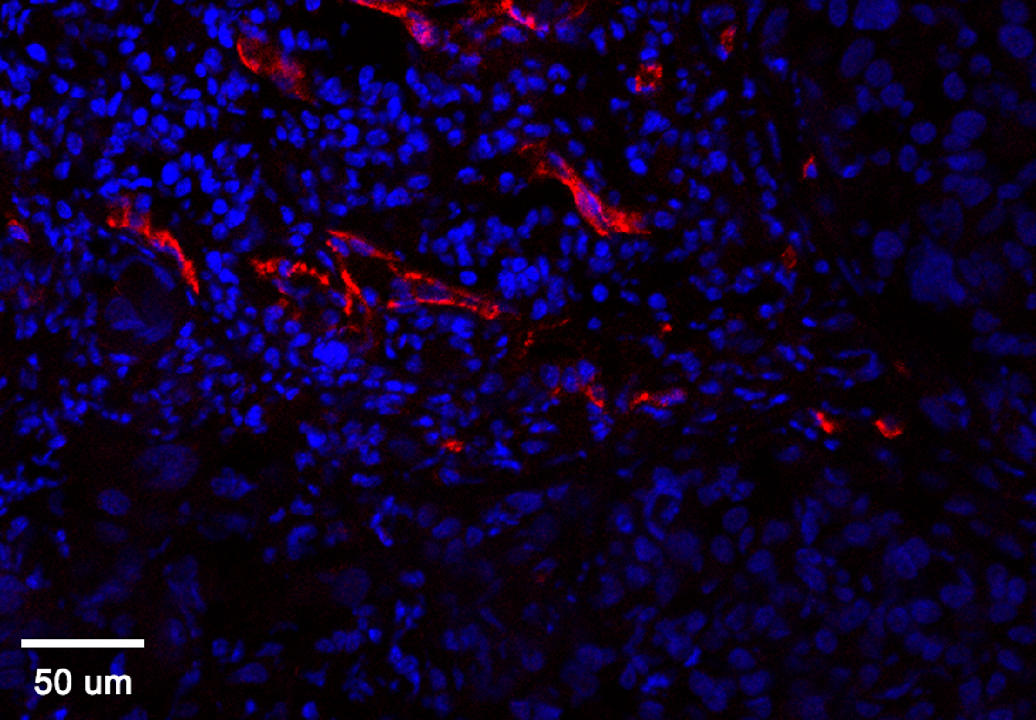

Supplement: S2 File — (ZIP) [file pone.0290861.s002.zip › S2 File/A group_Day0.tif]

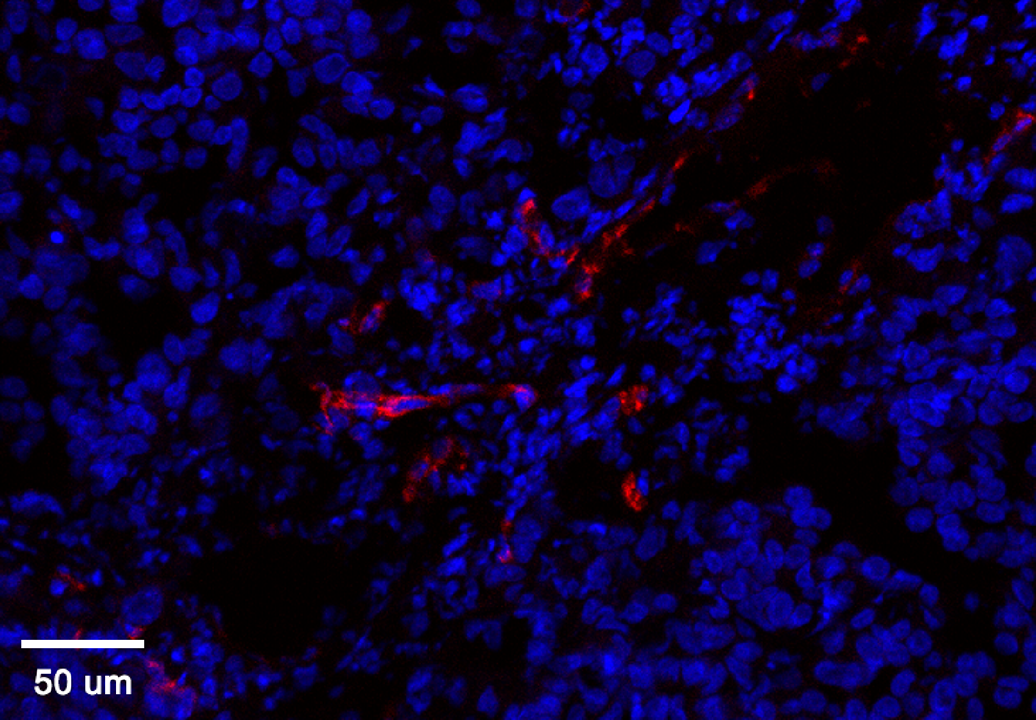

Supplement: S2 File — (ZIP) [file pone.0290861.s002.zip › S2 File/A group_Day14.tif]

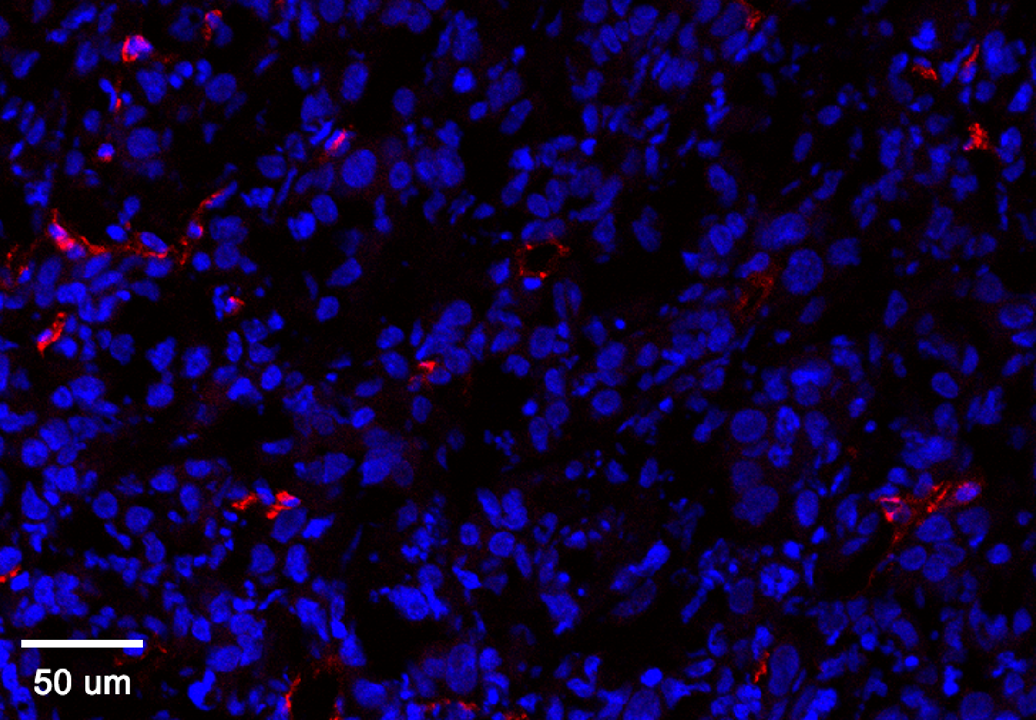

Supplement: S2 File — (ZIP) [file pone.0290861.s002.zip › S2 File/A group_Day21.tif]

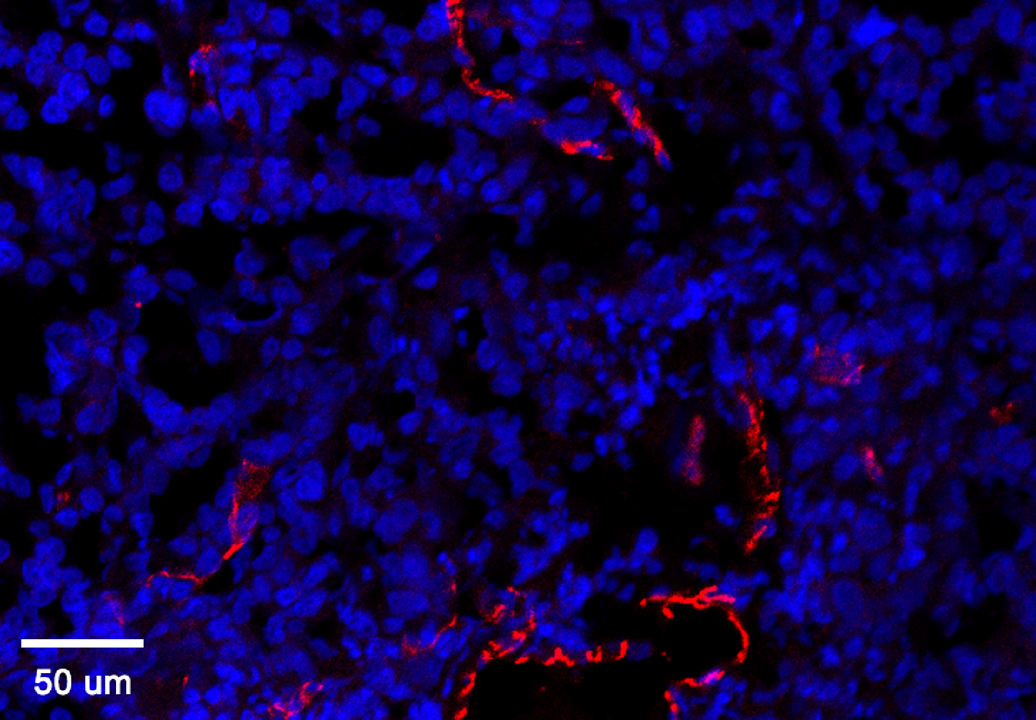

Supplement: S2 File — (ZIP) [file pone.0290861.s002.zip › S2 File/A group_Day3.tif]

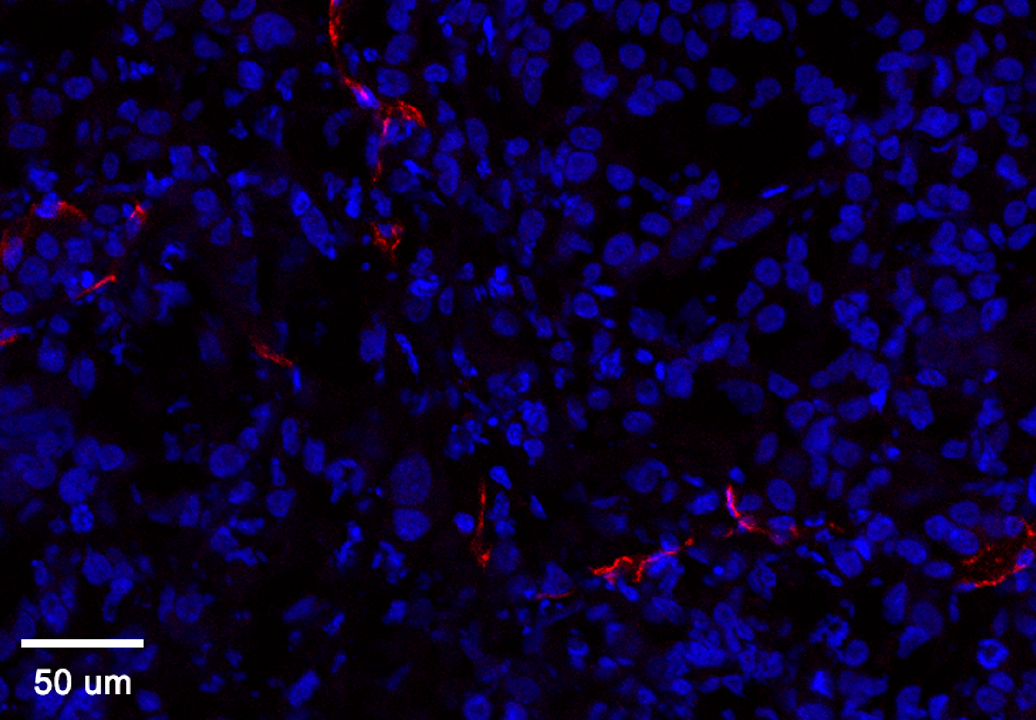

Supplement: S2 File — (ZIP) [file pone.0290861.s002.zip › S2 File/A group_Day7.tif]

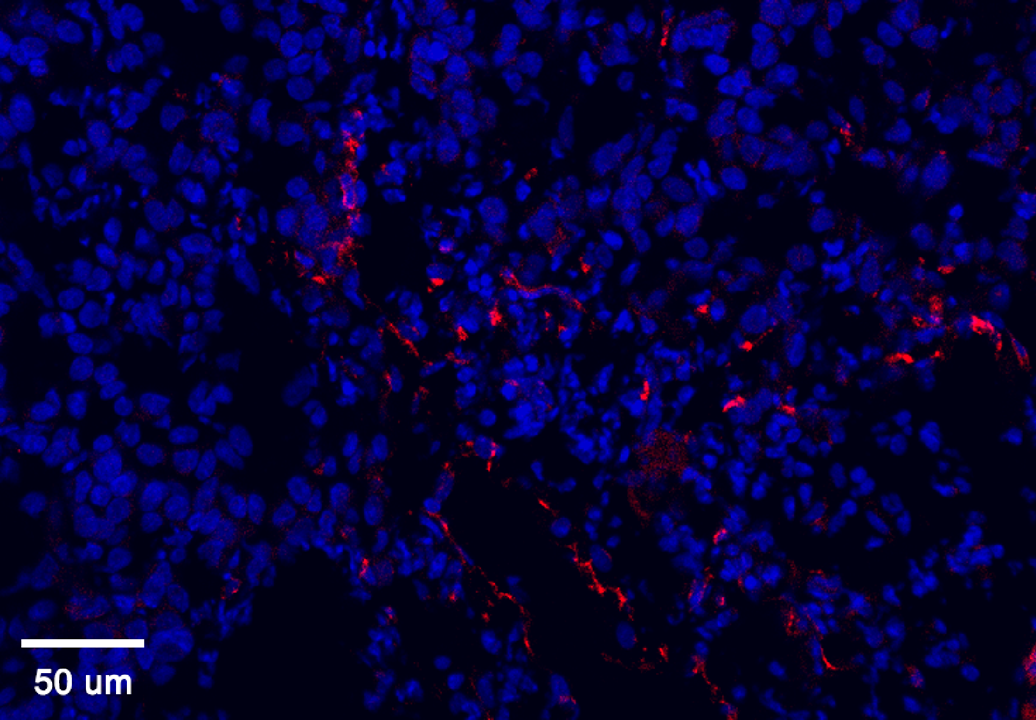

Supplement: S2 File — (ZIP) [file pone.0290861.s002.zip › S2 File/A-P group_Day0.tif]

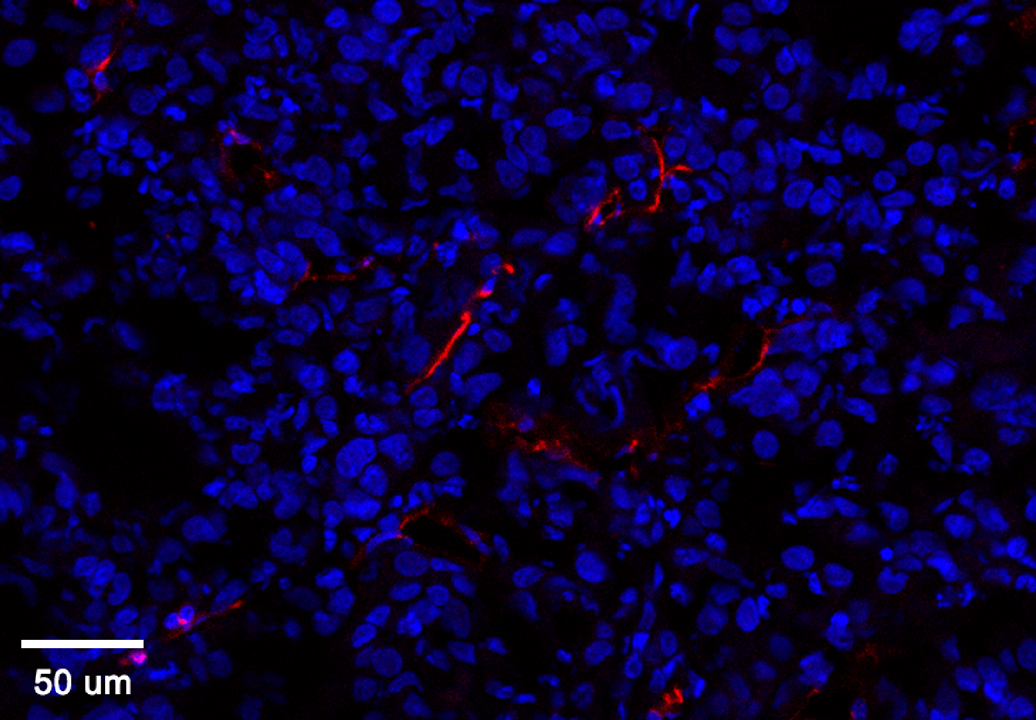

Supplement: S2 File — (ZIP) [file pone.0290861.s002.zip › S2 File/A-P group_Day14.tif]

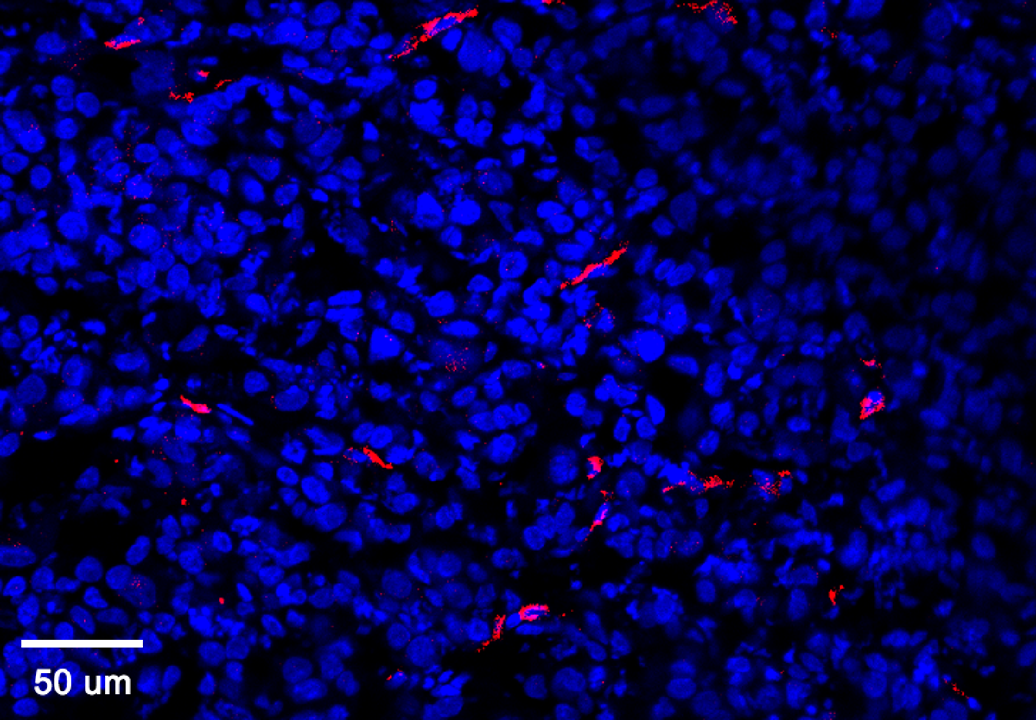

Supplement: S2 File — (ZIP) [file pone.0290861.s002.zip › S2 File/A-P group_Day21.tif]

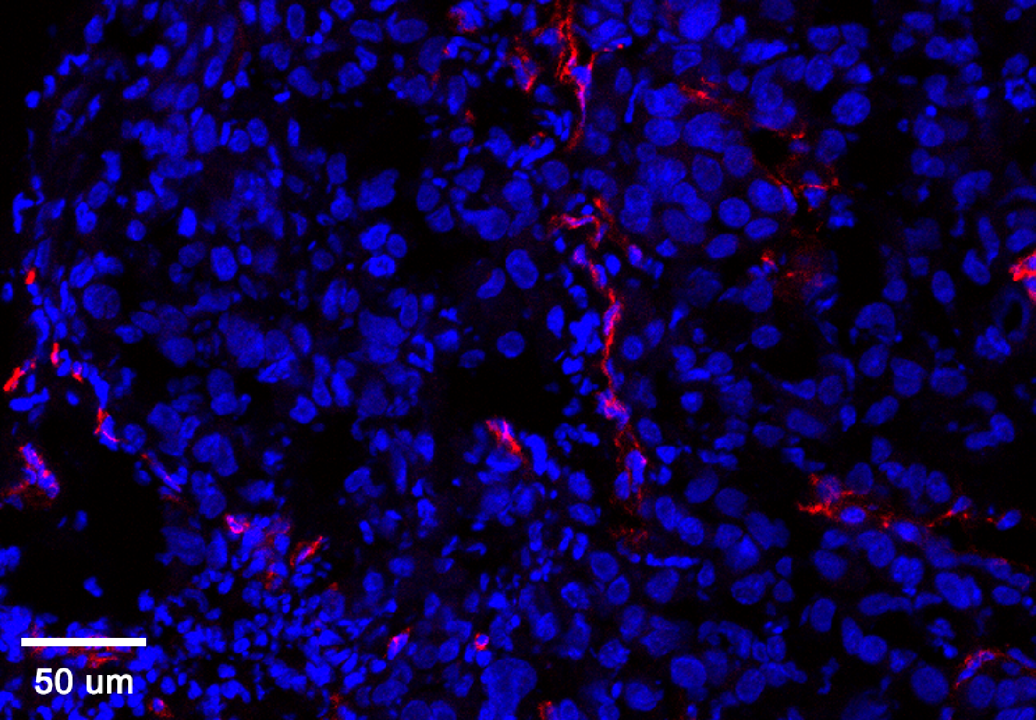

Supplement: S2 File — (ZIP) [file pone.0290861.s002.zip › S2 File/A-P group_Day3.tif]

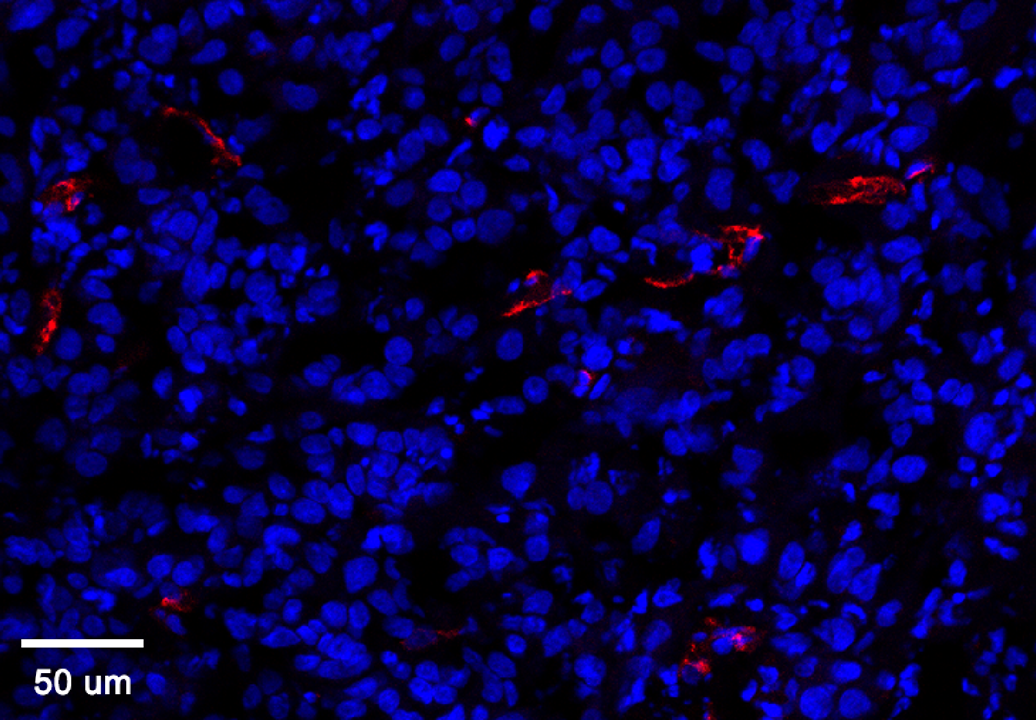

Supplement: S2 File — (ZIP) [file pone.0290861.s002.zip › S2 File/A-P group_Day7.tif]

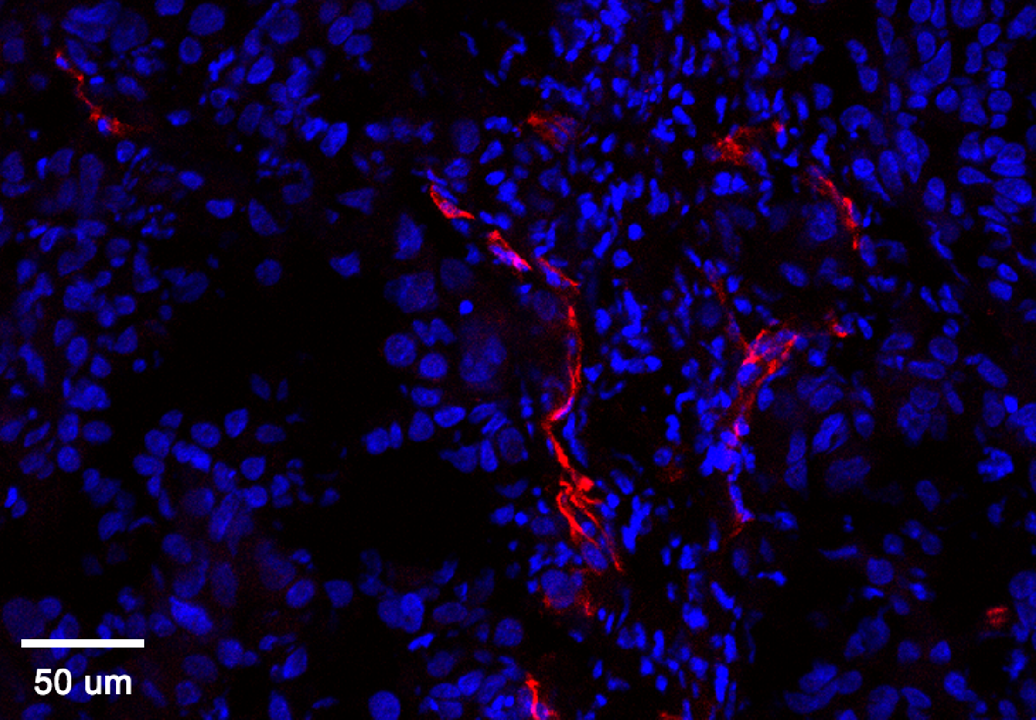

Supplement: S2 File — (ZIP) [file pone.0290861.s002.zip › S2 File/C group_Day0.tif]

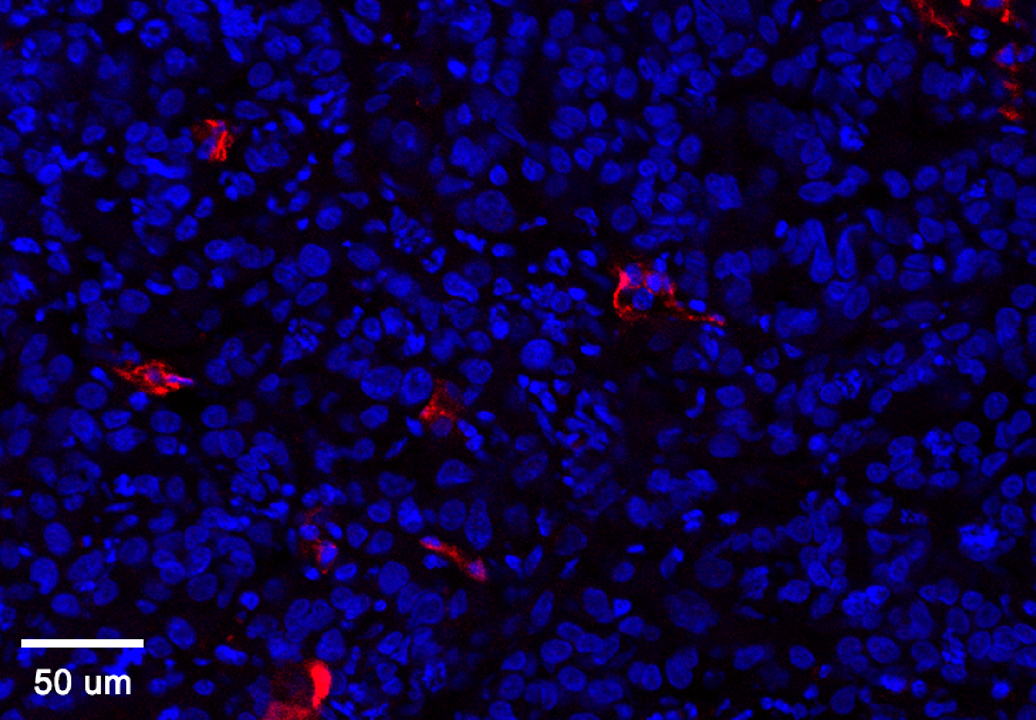

Supplement: S2 File — (ZIP) [file pone.0290861.s002.zip › S2 File/C group_Day14.tif]

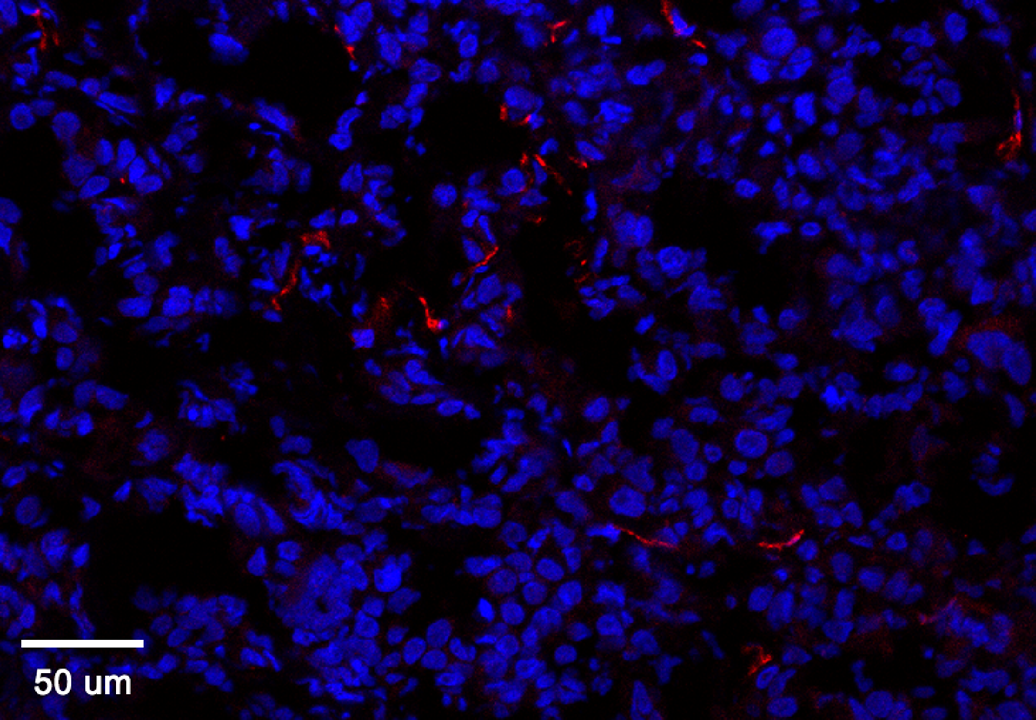

Supplement: S2 File — (ZIP) [file pone.0290861.s002.zip › S2 File/C group_Day21.tif]

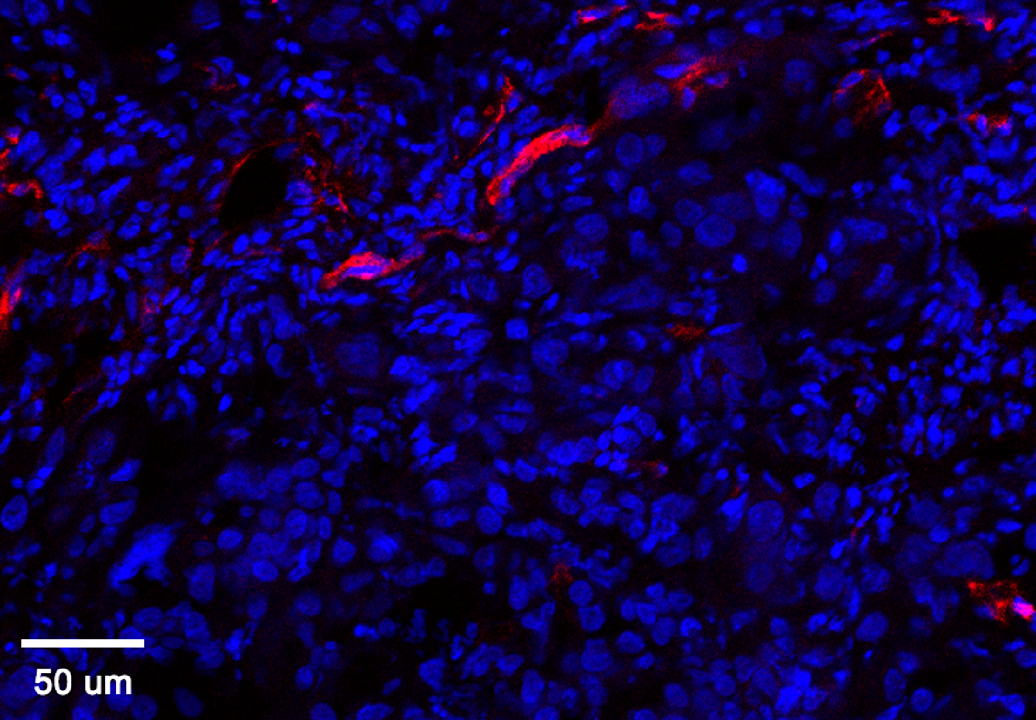

Supplement: S2 File — (ZIP) [file pone.0290861.s002.zip › S2 File/C group_Day3.tif]

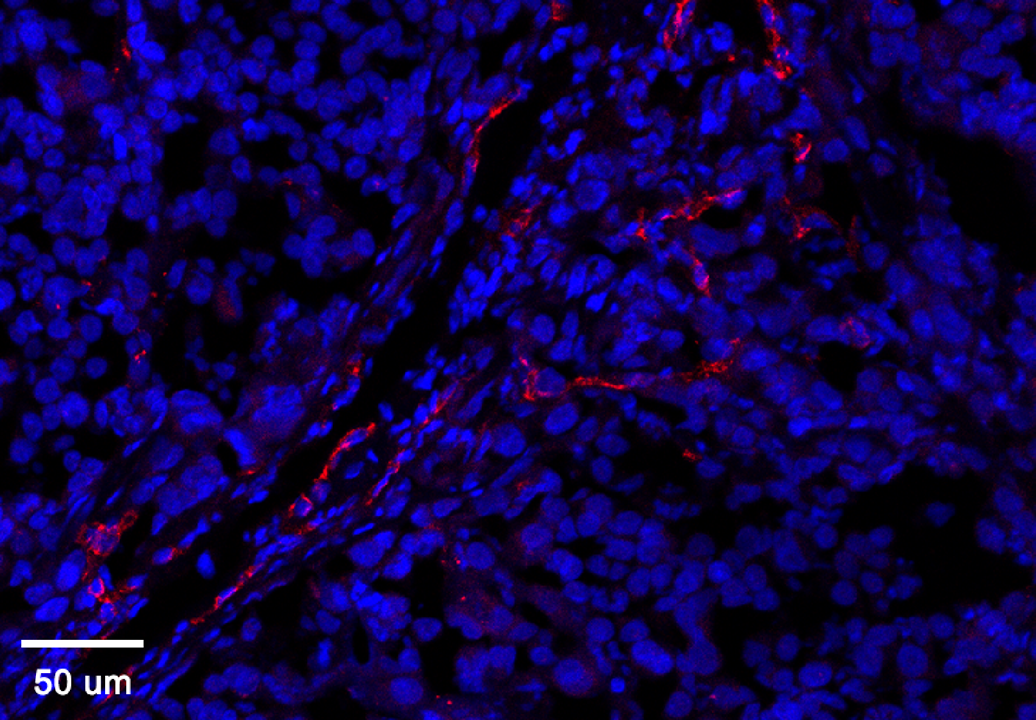

Supplement: S2 File — (ZIP) [file pone.0290861.s002.zip › S2 File/C group_Day7.tif]
