# Supplementary material for: Correction: DCE-MRI-Derived Parameters in Evaluating Abraxane-Induced Early Vascular Response and the Effectiveness of Its Synergistic Interaction with Cisplatin
Source: PLoS One. 2023 Aug 25;18(8):e0290861. doi: 10.1371/journal.pone.0290861 (PMC10456182; doi:10.1371/journal.pone.0290861)
Supplement: S1 Table — (DOCX) [file pone.0290861.s004.docx]

**Supplementary Table 1.** **Experimental Design for DCE-MR Imaging of Treatment Efficacy and Ex Vivo Histopathology**

|  | day | | | | | | | | |
| --- | --- | --- | --- | --- | --- | --- | --- | --- | --- |
| Parameter | 0 | 1 | 2 | 3 | 4 | 5 | 7 | 14 | 21 |
| C group | *,# |  | * | # | * |  |  |  |  |
| A group | +,# |  | + | # | + |  |  |  |  |
| A-P group | +,# | - | + | -,# | + | - |  |  |  |
| Histology | & |  |  | & |  |  | & | & | & |

“*” = PBS; “+” = Abraxane treatment; “-” = Cisplatin treatment; “#” = DCE-MRI; “&” = tumor tissue sampling.
